# Properties of Water Bound in Hydrogels

**DOI:** 10.3390/gels3040037

**Published:** 2017-10-19

**Authors:** Vladimir M. Gun’ko, Irina N. Savina, Sergey V. Mikhalovsky

**Affiliations:** 1 Chuiko Institute of Surface Chemistry, 17 General Naumov Street, 03164 Kyiv, Ukraine; vlad_gunko@ukr.net; 2 School of Pharmacy & Biomolecular Sciences, University of Brighton, BN2 4GJ Brighton, UK; I.N.Savina@brighton.ac.uk

**Keywords:** hydrogels, cryogels, strongly and weakly bound water, strongly and weakly associated water, interfacial phenomena, freezing-melting point depression

## Abstract

In this review, the importance of water in hydrogel (HG) properties and structure is analyzed. A variety of methods such as ^1^H NMR (nuclear magnetic resonance), DSC (differential scanning calorimetry), XRD (X-ray powder diffraction), dielectric relaxation spectroscopy, thermally stimulated depolarization current, quasi-elastic neutron scattering, rheometry, diffusion, adsorption, infrared spectroscopy are used to study water in HG. The state of HG water is rather non-uniform. According to thermodynamic features of water in HG, some of it is non-freezing and strongly bound, another fraction is freezing and weakly bound, and the third fraction is non-bound, free water freezing at 0 °C. According to structural features of water in HG, it can be divided into two fractions with strongly associated and weakly associated waters. The properties of the water in HG depend also on the amounts and types of solutes, pH, salinity, structural features of HG functionalities.

## 1. Introduction

Polar, hydrophilic natural and synthetic polymers physically or chemically cross-linked into 3D-network and bonding a large amount of water (up to 100 g/g or higher), but insoluble in water are known as hydrogels (HG, water containing gels). The insolubility of HG in water is of importance for preservation of the system integrity. A simple way to solve this task is the use of chemically cross-linked polymers or macromolecules, and the degree of cross-linking more strongly affects the behavior of HG in aqueous media than the behavior of water bound in HG. The HG hydrophilicity is owed to a number of water-solubilizing groups: –OH, –COOH, –COO^−^, >C=O, >CHNH_2_, –CONH_2_, –SO_3_H, etc., in the polymer network. Water plays an important role in hydrogels by supporting their integrity, solubility and diffusion of substances, which is important for biomedical, biotechnological and environmental applications [1,2,3,4,5,6,7]. The water content and therefore the HG volume can change due to swelling/shrinking influenced by external conditions, such as temperature, pH, ionic strength, solvent nature, etc.

The water in HG could be in different states: strongly (SBW) or weekly bound (WBW) to the polymer network; some amount of it behaves as free (non-bound) water (NBW). The water content changes in time because of the drying process. The analysis of the states of water in HG gives valuable information on their sorption and permeation properties, diffusion of water and solutes, as well as on the structure of pores filled with water. Here the properties of water bound in HG and interfacial phenomena in different natural and synthetic hydrophilic polymers and their composites with nanoparticles, i.e., nanocomposite HG, are analyzed. With changing temperature and hydration, polymer chains exhibit a complex hierarchy of dynamic processes, starting with very fast and local conformational rearrangements on the picosecond scale, and extending into the range of seconds for slow, diffusive and cooperative motions at *T* > *T*_g_ [8,9,10]. These processes are strongly influenced by water or/and solutes because of swelling, plasticizing, bonding, freezing-melting, and other effects. As a whole, the behavior of SBW and WBW (as well as NBW in macropores) and polymers at the interfaces of soft and solid materials depends on topology, topography, porosity, and surface chemistry of the materials, content and type of solvent or co-solvents and/or co-adsorbates and solutes, polymer polarity, charges, length, cross-linking degree, type and content of solid filler particles, temperature, etc. A number of experimental methods such as nuclear magnetic resonance (NMR) spectroscopy, differential scanning calorimetry (DSC), dielectric relaxation spectroscopy (DRS), thermally stimulated depolarization current (TSDC), microscopy, evaporation, thermogravimetric analysis (TGA), swelling, adsorption and diffusion of probe compounds, are used to characterize HG in the native state or treated by different methods (dried, heated, frozen, thawed, mechanochemically activated, hydrothermally treated, high-pressure treated, etc.). Clear, many of these effects are linked to the behavior of water being in different states.

Water in HG can be classified into several types: (i) bound water, which comprises strongly bound (SBW, unfrozen at *T* < 260–265 K), weakly bound (WBW, unfrozen at 260–265 K < *T* < 273 K), and non-bound with nearly bulk water properties (NBW, frozen at 273 K); (ii) associated water, which comprises strongly associated (SAW, chemical shift δ_H_ > 2 ppm, with 3D clusters and domains of various sizes) and weakly associated water (WAW, δ_H_ = 1–2 ppm, 1D–2D clusters or alone molecules in hydrophobic surroundings); and (iii) free water [11,12,13]. SBW corresponds to non-freezing bound water, and WBW corresponds to freezing bound water as derived from DSC data (*vide infra*). Not only the type of water binding but also its activity as a solvent, local (nanoclusters, nanodomains) and micro-scaled (microdomains) structure, density, electroconductivity, and other properties influence the properties of HG [11,12,13,14,15,16,17,18,19,20,21]. Here the relationships between bound water organization and its properties linked to the properties of HG will be analyzed using data obtained by several experimental methods (NMR, DSC, TSDC, DRS, diffusion, etc.) and theoretical modeling.

## 2. Structures of Hydrogels and Cryogels and Characteristics of Bound Water

### 2.1. HG with Synthetic Polymers and Polysaccharides

Many physical properties of HG depend on the organization of water within and at the surface of HG [22]. According to phase transition behavior, three types of water in HG have been identified: non-freezing (i.e., SBW), freezing bound (WBW and a small fraction of SBW) and free (NBW) water [23]. Melting, crystallization, and glass transition of water in HG reflects the state of the water interacting with polar 3D network, e.g., with polysaccharides or other polar polymers. Freezing bound water forms metastable ice by slow cooling and amorphous ice by quenching in contrast to free (NBW) water in HG forming the stable hexagonal ice. From the isothermal crystallization measurements, nucleation rate and crystal growth rate could be estimated, and the crystal growth rate of freezing bound water is about ten times slower than that of free water. Ice nucleation in SBW and WBW is shifted toward lower temperatures (lower for SBW) due to freezing point depression described by the Gibbs-Thomson relation [13]. A decrease in the water content changes the relationship between the starting melting temperature and crystallization temperature. Both temperatures decrease with decreasing degree of hydration [23] that can be explained by increased fraction of SBW.

Solute diffusion, adsorption, release, etc. in HG is important in many biotechnology fields. Solute behavior in HG is determined by different factors such as HG free volume (e.g., macroporosity and interconnectivity of macropores), hydrodynamic drag on the solute, increased path length due to obstruction, and a combination of hydrodynamic drag and obstruction effects of the polymer matrix [24]. Several mathematical models have been derived to explain and predict solute diffusion in HG. These models can be divided into those applicable to HG composed of flexible polymer chains and those composed of rigid polymer chains. For HG with flexible polymer chains, it was determined that a scaling hydrodynamic model provided the best explanation for solute diffusion, while for HG with rigid polymer chains obstruction models were more consistent with the experimental data. Both the scaling hydrodynamic model and the most appropriate obstruction model contain undefined parameters, which should be clarified in order for these models to gain widespread acceptance [24]. However, this model ignores such factor as the porosity of the macropore walls, which can play an important role for diffusion and adsorption of not only low-molecular weight solutes but also high-molecular weight ones, such as proteins (*vide infra*) [13].

Various synthetic and biopolymers, proteins and other macromolecules can be used as the HG matrices in biomedical and pharmaceutical applications because of their high biocompatibility and hydrophilic nature [25]. The organization of water bound in these materials is strongly dependent on the surroundings (e.g., HG porosity) and external factors [11,12,13,14]. Biocompatible highly porous HG with pore size *d* > 1 μm have potential for biomedical applications such as tissue regeneration [26], and these applications are mainly defined by two factors: state of water inside HG and the pore structure of the HG, including pores in the macropore walls, allowing effective mass transport of solutes. The water content in gels could vary in a very wide range. Those HG which uptake very high amounts of water are known as super-swelling gels. Some hydrogels based on homopolypeptides, such as poly(aspartic acid), poly(glutamic acid), and poly(ε-l-lysine) have been reported as having super-swelling properties [27].

The states of water in poly(vinyl alcohol) (PVA) based HG were analyzed at time scales of a few nanoseconds [28], however some conclusions were controversial. Several factors should be taken into account to interpret the experimental data regarding the states of water in HG. Firstly, it has to be determined whether water in HG is found in different dynamic states or whether there is just a single albeit broad distribution of characteristic times. The response depends on the dynamic process used as a probe and, hence, on the experimental method used [28]. Secondly, at least, two types of bound water could be distinguished in the temperature range below the normal freezing point of bulk water. Therefore, while it is correct to determine different fractions of water undergoing phase transitions at different temperatures during DSC experiments (or low-temperature other ones), the concept of different states of water should not be applied to HG above 273 K (however, some methods, e.g., TG, FTIR, etc. can give information on water states at *T* > 273 K [13]). Thirdly, not necessarily “free” water molecules are more mobile than the “bound” molecules, as it is widely believed [28]. The data based on TSDC, NMR, and DRS measurements related to direct current (dc) relaxations and water molecules mobility suggest that despite reduced mobility of bound molecules they remain mobile at *T* < *T*_f_ (freezing point) [11,12,13]. Bound water can be more mobile (e.g., in rotation) at *T* < 273 K than free water transformed into ice, in which proton transfer mainly due to tunneling effect could be observed at low temperatures.

A molecular dynamic (MD) model was used to study PVA HG cross-linked by radiation [29]. The results of MD simulation showed good agreement with the experimental data. It was shown that in a small range of cross-linking degree, the content of non-freezing water (i.e., SBW) in PVA HG remains practically constant in contrast to WBW and NBW [29]. MD simulations were performed for aqueous solutions of PVA, poly(vinylmethylether) (PVME), and poly(*N*-isopropylacrylamide) (PNiPAM) [30]. The distributions and dynamics of hydrogen bonds, the translational diffusion of water, and the orientational relaxation of water were analyzed to investigate the properties of water influenced by the polymer chains. The water molecules around the polymer chains are highly hindered by the polymers. The water molecules were classified into three categories: (1) those around hydrophilic groups (maximal for PVA with COH), (2) those around hydrophobic groups (maximal for PVME), and (3) bulk region (outside the regions 1 and 2) (maximal for PVA due to strong hydrogen bonds between COH groups of PVA). The orientational relaxation time of water becomes long because of formation of hydrogen bonds between water and the hydrophilic groups of polymer chains and structuralization of water around the hydrophobic groups.

The states of water within HG prepared from diepoxy-terminated poly(ethylene glycol)s (PEG) of different molecular weights cross-linked with various aliphatic polyamines were studied using DSC [31]. PEG molecular weight, weight composition of the (α,ω-dihydroxy PEG) DEPEG_600_-DEPEG_4000_ mixtures, amine chain length, amine functionality and structure and amine/epoxy groups molar ratio upon the different types of water in HG affected the water properties. It was shown that the amount of freezing water depended mostly on the structure and size of the meshes of the polymer network, while the non-freezing water content was mainly affected by the chemical structure of the network [31]. This is due to the fact that the first water type is WBW, whereas the second one is SBW located closely to the polymer functionalities.

The influence of the polymer side chain structure on the behavior of water bound in hydrogels was studied using Raman spectra of polyacrylamide (PAAm) and poly-*N*,*N*′-dimethylacrylamide (PDMAAm) HG [32]. These polymers have similar backbone structures, except for the side chain. The frequency of the O–H stretching vibrations correlates with water content in PAAm, but it increases in PDMAAm upon decreasing water content. From the comparison of the relative intensities of the O–H stretching vibrations, the water density appeared to be different in PAAm and PDMAAm hydrogels. This difference can be explained by the nature of bonds between tetragonal water structures and the side functional groups in the polymer hydrogels. Whereas water clusters form strong hydrogen bonds with the hydrophilic groups of PAAm, the side functionalities in the PDMAAm form weak hydrogen bonds with water molecules [32].

To investigate the mechanisms of structural changes in polymer network and water during dehydration, X-ray diffraction of poly-*N*,*N*′-dimethylacrylamide HG was measured [33]. The individual structures of water and PDMAAm were analyzed by decomposition of the diffraction patterns to separate their respective contributions. The results showed that the short-range structures of PDMAAm expand during dehydration, whereas the network structure as a whole shrinks. The average length of the hydrogen bonds between water molecules increases during the dehydration process [33]. This can be explained by maximal distortion of the hydrogen network in SBW, whose fraction increases with dehydration, since NBW and WBW are removed before SBW.

The swelling properties of polyacrylic acid (PAA) hydrogel beads were studied vs. time and temperature [34]. The bead size, swelling capacity, water retention, swelling ratio and degree were analyzed to describe the swelling properties of PAA HG. The maximum swelling ratio of 233.7 was achieved at 40 °C. The swelling process was characterized by non-Fickian diffusion [34]. Clearly, in strongly swollen polymers, contribution of NBW and WBW is much larger than that of SBW. There is a consequence of this effect such as stronger damage of strongly swollen HG at *T* < 273 K in comparison with weakly hydrated systems.

Ionic HG were used to design intelligent drug releasing systems. In particular, anionic hydrogels were tested for protein delivery in the large intestine, to prolong their bio-activity. Cationic hydrogels were used to develop smart insulin delivery system, which are sensitive to glucose concentration [35]. The effect of the methods of HG synthesis on the mechanism of water transport through the ionic hydrogels and the release mechanism of a solute from the HG, was studied. The diffusion coefficient of water and solutes depended on the content of methacrylic acid responsible for the negative charge in the anionic HG.

The enthalpies of water melting (Δ*H*_m_) and absorption (Δ*H*_wa_) in poly(methacrylic acid) (PMAA) and copolymers of MAA with hydroxyethylmethacrylate (HEMA) were estimated in [36]. The value of Δ*H*_m_ of water bound in HG increased with increasing equilibration time at −15 °C. However, it remained smaller than Δ*H*_m_ of pure water due to the clusterization of bound water [13]. The value of Δ*H*_wa_ was negative and decreased with increasing initial amount of water in the HG. However, the observed increase of Δ*H*_m_ was not fully compensated by the decrease of Δ*H*_wa_. This effect is probably due to the fraction of water which remained unfrozen during the cooling-heating cycle. The glass transition temperature (*T*_g_) of HG decreased upon hydration. The amount of water required to decrease *T*_g_ to 0 °C approximately corresponded to the amount of unfrozen water bound to the HG. Thus, the amount of non-freezable structured water (i.e., SBW) cannot be explained by the existence of various types of water. It is related to the restriction both of the water diffusion and further ice crystal growth after the hydrogel transition from the rubbery state to the glassy state which is characterized by the loss of flexibility of the polymer network. Similar effects lead to the Gibbs-Thomson effect of the freezing-melting point depression of liquids under confined space effects [13].

The effect of chitosan/polyvinyl alcohol (Ch-PVA) molar ratio, concentration of the cross-linker glutaraldehyde (*C*_GA_), and the ionization state of the polymer matrix on the amount of bound water (X_BW_) was studied in the novel pH-sensitive, biodegradable Ch-PVA hydrogel [37]. DSC was used to measure X_BW_ in the initial HG, and in the hydrogel in pH 3 and pH 7 buffers. In the initial HG, X_BW_ increased with increasing PVA concentration (*C*_PVA_), but it was not significantly affected by *C*_GA_. In other words, the degree of cross-linking weakly affects the bound water state and behavior. In the buffer-equilibrated HG, X_BW_ showed a different trend decreasing with increasing *C*_PVA_ and decreasing *C*_GA_. The amount of bound water calculated per unit of hydrogel mass (*C*_BW_) was significantly higher in the ionized (swollen) HG than in its non-ionized state. This can be explained by the association of large water clusters with the −NH_3_^+^ groups of chitosan, when the gel was swollen in the acidic medium. The value of *C*_BW_ is maximal (ca. 3.5 g/g) at pH 3 and strongly decreases at pH > 5 from 1.5 (pH 5) to 0.2–0.3 g/g at pH 10–11.

Modified chitosan HG membranes were prepared using GA and sodium citrate (CIT) as cross-linking agents [38]. It was found that cross-linking influenced both molecular and supramolecular structure of membranes as well as swelling properties and state of water in the membranes studied. The equilibrium water content decreased in the following order: Ch > Ch/GA > Ch/GA/CIT. DSC studies showed the presence of both freezing (NBW, SBW) and non-freezing (SBW) water in non-cross-linked and cross-linked chitosan membranes. The formation of different states of water within a polymeric network took place in the following order: non-freezing, freezing bound and freezing free water. For all membranes, the freezable water content increased linearly with the water uptake and the non-freezable water content remained constant beyond a certain critical value (ranging from 0.47 to 0.65 g/g dry membrane). These values characterize the amounts of water located in narrow pores (voids).

Spin-lattice and spin-spin relaxation in chitosan HG was studied at 200–320 K [39]. Spin-lattice relaxation time *T*_1_ was measured using an aperiodic saturation recovery sequence while a CPMG pulse sequence was used to measure spin-spin relaxation time *T*_2_. At a low degree of cross-linking, chitosan (Ch) forms superabsorbing HG. A very high water content in the fully swollen hydrogels masks the properties of bound water. To distinguish the bound water, dried Ch and its cross-linked derivatives were rehydrated stepwise to analyze the relaxation effects vs. temperature and water content. The number of water molecules in the solvation shells of chitosan was estimated from ^1^H NMR relaxation. While about four H_2_O molecules per repeat unit of the macromolecule were tightly bound (SBW) in chitosan, their number increased in cross-linked Ch (this is rather effect of confined space enhancing contribution of SBW). It correlated with the swelling properties of the network. Most theoretical models of HG account for diffusion both for homogeneous and heterogeneous systems. However, there is no clear distinction between these systems. NMR relaxation studies contributed to a more clear distinction between the homogeneity and heterogeneity of hydrogels prior to gel formation [39].

To study the intrinsic heterogeneity of polysaccharide hydrogels and dynamic processes such as segmental motions of the polymer chain and molecular diffusion in HG a number of experimental techniques and methods have been used [40]. They included well-known techniques such as measurements of swelling and elastic modulus, and emerging methodologies in the field such as elastic and quasi-elastic neutron scattering (QENS), and fluorescence recovery after photobleaching. The dynamic processes in polysaccharide HG occur on a wide time scale, which creates significant problems in the choice of the experimental techniques. The strong coupling between the processes makes the studying of the dynamic behavior of these materials a difficult task. The study of dynamic processes in HG requires the use of complementary methods with appropriate temporal and spatial resolution, which do not strongly perturb the materials. Applying QENS and NMR relaxometry, the dynamics of protons belonging to both the polymer structures and water can be studied. QENS method probes dynamic events occurring in pico- to nanosecond time ranges, which are typical for molecular dynamics (MD) simulations. Thus, the experimentally determined diffusion coefficients of protons belonging to confined water in the HG or to the chain segments can be compared with the values derived from MD simulations. Combining novel spectroscopic techniques designed or developed for soft materials with equilibrium thermodynamics, NMR relaxometry, and rheology provides comprehensive background to studying dynamic processes in hydrogels [40].

A series of HG was synthesized by cross-linking natural or semi-synthetic polysaccharides such as carboxymethylcellulose, hyaluronic acid and chitosan. The cross-linkers were chosen according to the chemical structure of the polymer chains [41]. Bound water was found to be responsible for the injectability of hydrogels, an important characteristic of HG determining their suitability for mini-invasive surgery and localized therapy. It was found that under a shear stress (e.g., upon passage through a syringe needle), the water molecules could convert from a bound state to a semi-bound state. This, in turn, could decrease the mechanical parameters of HG and increase the degree of swelling [41].

The effects of a type of substituent of the cellulose ethers (such as hydroxyethyl cellulose (HEC), hydroxypropyl cellulose (HPC), and hydroxypropyl methyl cellulose (HPMC) and the molecular weight of the polymer on the state and dynamics of water bound in the HG were analyzed in [42]. To measure the amount of polymer bound water and thus to calculate the average number of water molecules bound to a polymer repeat unit (PRU), the ^1^H NMR spectroscopy was used. The values of ^1^H NMR *T*_1_ and *T*_2_ of water in HG at different cellulose ether concentrations, *C*_pol_, at room temperature decreased with increasing polymer concentration. *T*_1_ decreased approximately two-fold with increasing *C*_pol_ from 0 to 10 wt %, the *T*_2_ value decreased by an order of magnitude and the number of water molecules bound per PRU (*n*_bw_) increased from 2–3% to 9–16% at *C*_pol_ ≈ 40 wt %. The relaxation rate 1/*T*_1_ was sensitive to the nature of polymer substituent, but not to the molecular mass of the polymer. On the contrary, the rate 1/*T*_2_ was weakly influenced by the polymer substitution. Based on the analysis of the *T*_1_ and *T*_2_ data, *n*_bw_ was found to be the largest for HPC followed by HEC and HPMC. These results correlated with the degree of hydrophilic substitution in the polymer chains. Since the ^1^H NMR relaxation technique probed a single molecular layer of bound water in the polymers at all concentrations, it can be concluded that the mesh size of polymer network did not change [42].

The water content in different states can affect interactions of solutes, large bio-molecules and cells with polymers, especially within nanopores in macropore walls, micro- and macropores. According to the classification of pores suggested for porous polymers used in tissue scaffolds, (cylindrical) pores with diameter *d* < 0.1 μm, 0.1 μm < *d* < 100 μm, and *d* > 100 μm are defined as nano-, micro-, and macropores, respectively [43]. Nanopores can be further divided into three sub-types such as narrow (*d* < 2 nm), middle (2 nm < *d* < 50 nm) and broad (50 nm < *d* < 100 nm) nanopores [44], that correspond to micro-, meso- and macropores, respectively, of the well-known IUPAC classification of pores [45,46]. The IUPAC classification is based on the different mechanisms of gas adsorption in pores vs. their sizes and although it is widely used to characterize adsorbents, it is not suitable to describe pores in materials used in biomedical applications particularly because the molecular size of solutes in liquid phase is much larger than molecules of gases. The determination of the amounts of different types of water in porous hydrogels is of importance for understanding the nature of adsorption/desorption processes in HG and assessment of their biocompatibility [25,26,44,47]. For these purposes, ^1^H NMR spectroscopy and DSC methods can be effectively applied in parallel [13].

### 2.2. HG Containing Proteins, Biopolymers and Synthetic Polymers

Experimental methods frequently used to characterize the texture of hard porous materials, such as gas/vapor adsorption, mercury porosimetry, pycnometry and conventional scanning electron microscopy, are not directly applicable to hydrogels (especially prepared with proteins or other biomacromolecules), which have to be dried and degassed [13]. However, drying and evacuation of HG almost certainly results in their shrinkage and deformation, closure or collapse of the porous structure of these soft materials. Therefore, nondestructive methods provide a more adequate analysis of the porous structure of hydrogels in hydrated state. Native or freeze-dried HG of poly(2-hydroxyethyl methacrylate-*co*-allyl glycidyl ether), HEMA-AGE, gelatin, G, and gelatin-fibronectin, G-Fn, were studied using SEM, cryo-SEM, confocal laser scanning microscopy (CLSM), and multiphoton microscopy (MPM) [47,48]. The microscopic images were analyzed using ImageJ [49] and Fiji software [50]. DSC measurements were performed on both hydrated and freeze-dried HG. The DSC thermoporometry was used to calculate the pore size distributions. The ^1^H NMR spectra recorded at 200–280 K allowed measuring the amount of unfrozen water. The molecular structure of the hydrogel fragments was calculated using the PM6 and PM7 semiempirical methods with the MOPAC 2012 and 2016 computational program suites [51].

The morphology of (HEMA-AGE) gels of various compositions was studied using CLSM and MPM, which reveal the morphology of hydrated native gels. The image analysis provides the data on the porosity, specific surface area, pore size and pore wall thickness (Table 1). Both CLSM and MPM give similar results considering certain limitations of image analysis using ImageJ and Fiji software [47,48].

**Figure 1:**
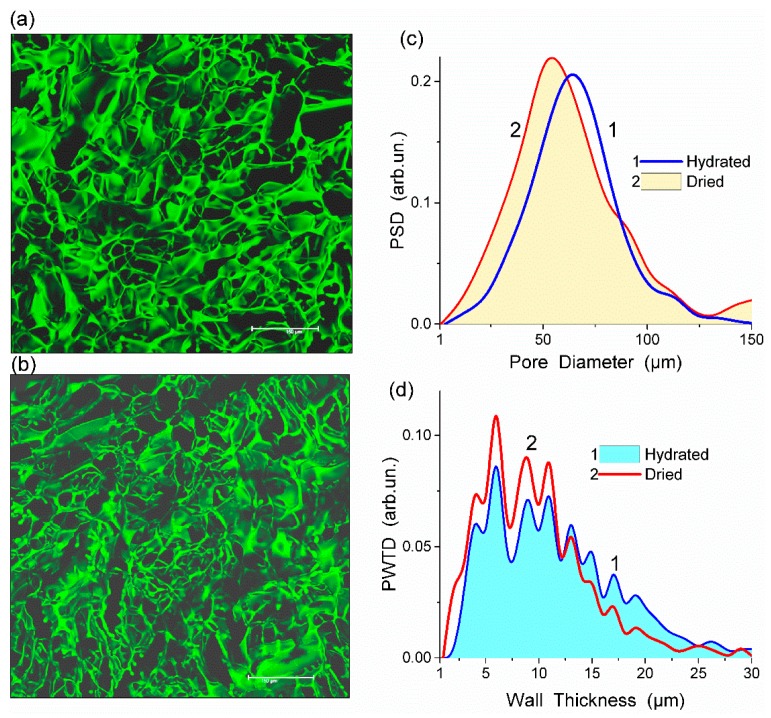
Confocal laser scanning microscopy (CLSM) images of HEMA-AGE hydrogel (sample A) in (**a**) hydrated and (**b**) dried states (scale bar 150 μm) with the pore (**c**) size and (**d**) wall thickness distributions (reproduced from Ref. [47] with permission from The Royal Society of Chemistry).

Fluorescein isothiocyanate (FITC) stained HEMA-AGE gel was freeze-dried and its CLSM image was compared with that of the hydrated sample (Figure 1a,b). This comparison shows that changes in the porous structure of HG after freeze-drying were insignificant (Figure 1c,d). The observed changes could be attributed to the shrinkage of polymer walls rather than shrinking and collapsing of macropores. During drying the mean thickness of the macropore walls decreased from 11.2 to 9.6 μm which is within the errors of the analysis (Table 1). A major part of water in HG is located in macropores rather than in the swollen pore walls. A relatively small thickness of the walls provides the HG elasticity, sponge-like morphology and significant mechanical strength.

**Figure 2:**
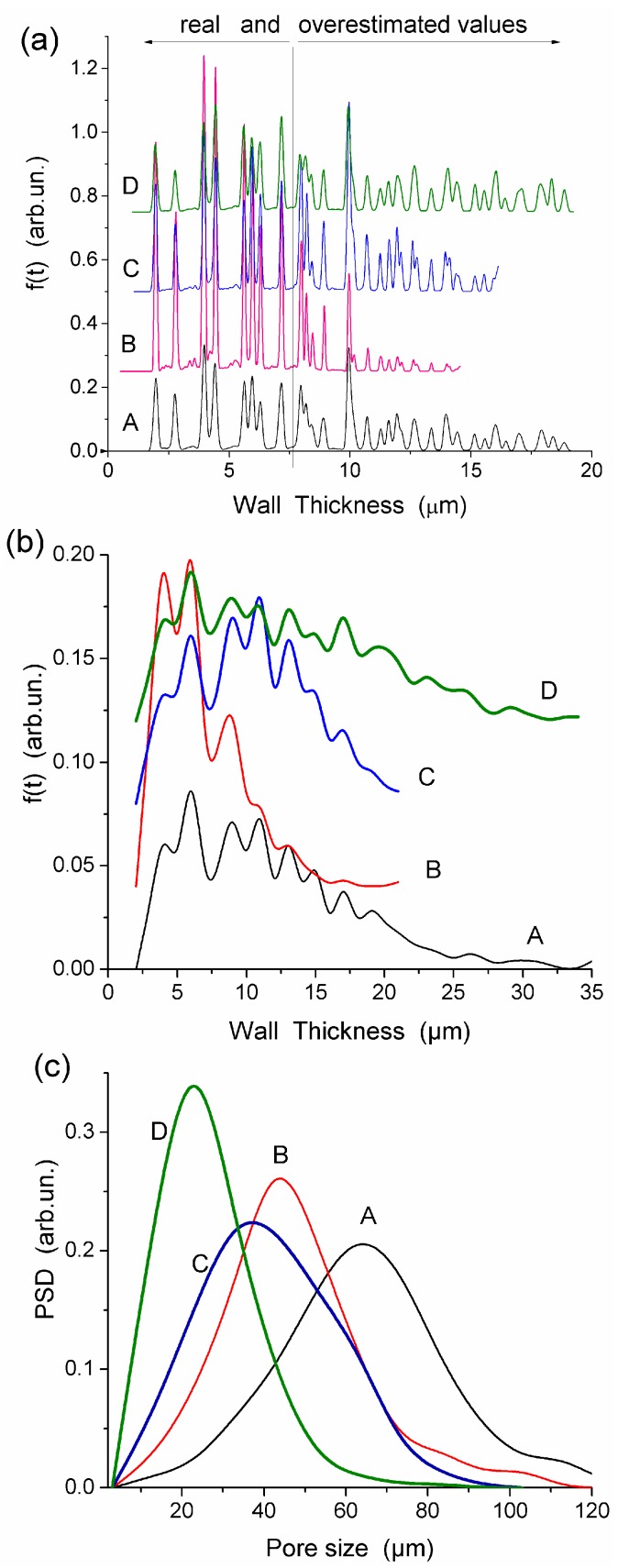
Wall thickness distributions with (**a**) Fiji and (**b**) ImageJ, and (**c**) pore size distributions for HEMA-AGE HG A, B, C and D (Table 1) (reproduced from Ref. [47] with permission from The Royal Society of Chemistry).

**Figure 3:**
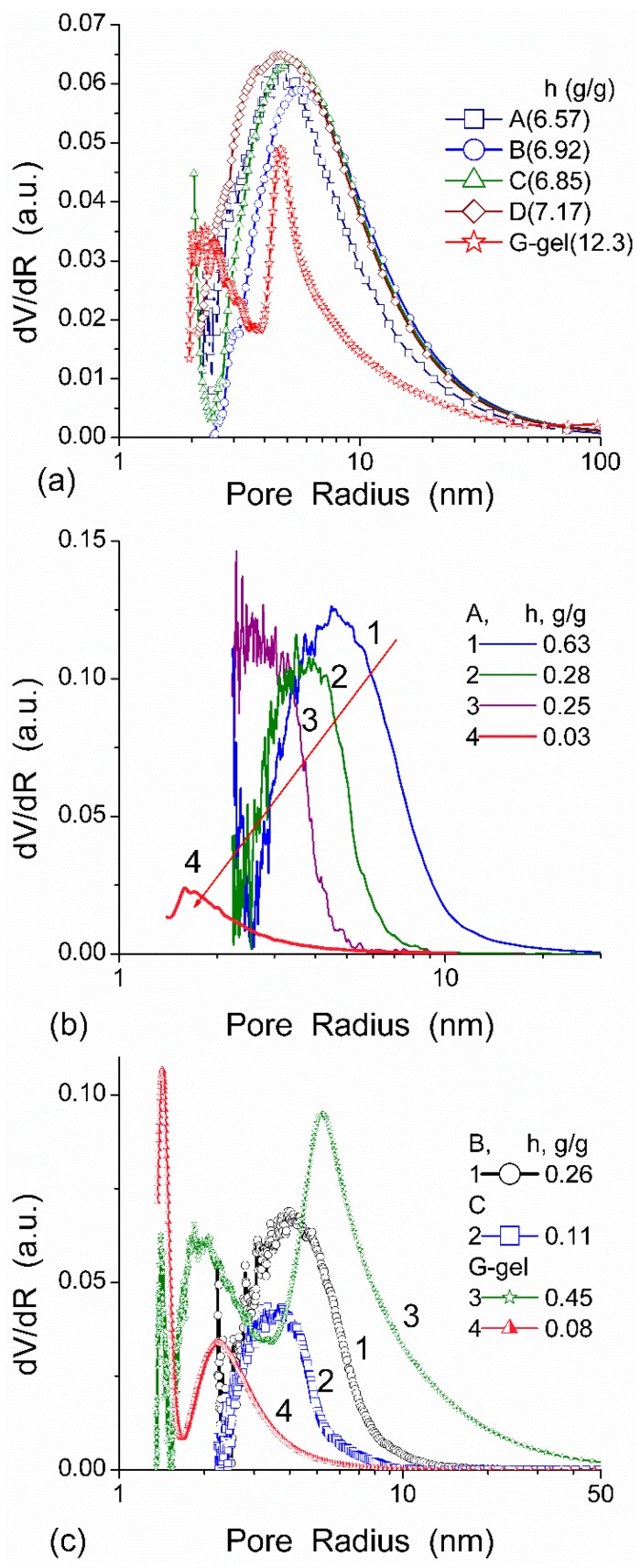
PSDs calculated from the DSC data for HEMA-AGE (A, B, C, and D samples) and G gels (hydration *h* = *m*_w_/*m*_d_ where *m*_w_ is the weight of water evaporated in DSC measurements up to 160 °C and *m*_d_ is the residual weight of heated sample) at (**a**) high and (**b**,**c**) low hydration (reproduced from Ref. [47] with permission from The Royal Society of Chemistry).

All HEMA-AGE gel samples had relatively uniform pore size and wall thickness distributions (Figure 2). The pore sizes of HG samples A, B and C were mainly in the range of 3–100 μm (Table 1). Gel D displayed a narrower pore size distribution (3–83 μm). The wall thickness distribution of gel B was narrower (2–14 μm) while the gels A, C and D had broader wall thickness distributions (2–32 μm, 2–23 μm and 2–32 μm, respectively) (Figure 2b). According to DSC thermoporometry data, samples A–D had relatively large specific surface area *S* ~80–90 m^2^/g because of the nanoporous structure of the macropore walls. Water was bound in the nanopores with the radius of 1–30 nm (Figure 3), which are located in the macropore walls of several μm in thickness (Figure 2).

**Figure 4:**
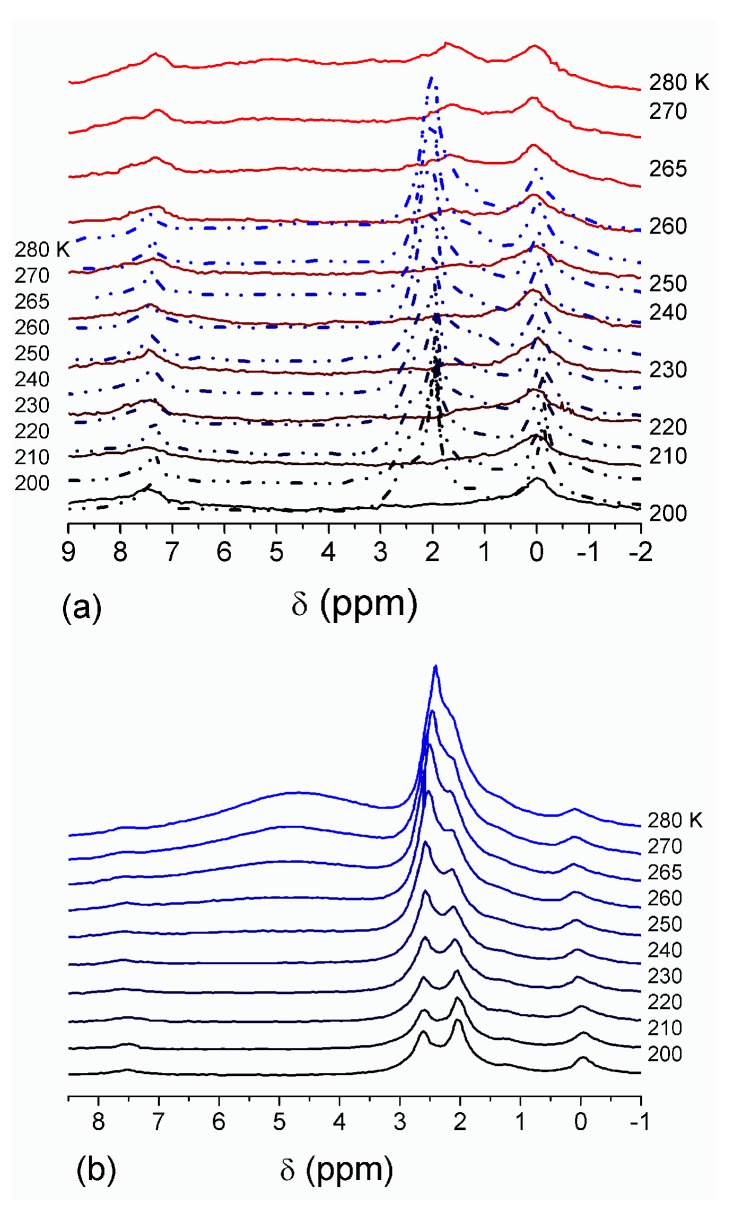
^1^H NMR spectra, of water adsorbed by gelatin gel recorded at different temperatures: (**a**) initial freeze-dried (0.3 wt % H_2_O) in CDCl_3_ (solid lines) and in a mixture CDCCl_3_:CD_3_CN 3:1 at (**a**, dashed-dotted lines) 0.8 wt % and (**b**) 10 wt % of water. Signal at 0 ppm corresponds to tetramethylsilane added as a standard; signal at 7.2 ppm corresponds to residual CHCl_3_ (reproduced from Ref. [47] with permission from The Royal Society of Chemistry).

**Figure 5:**
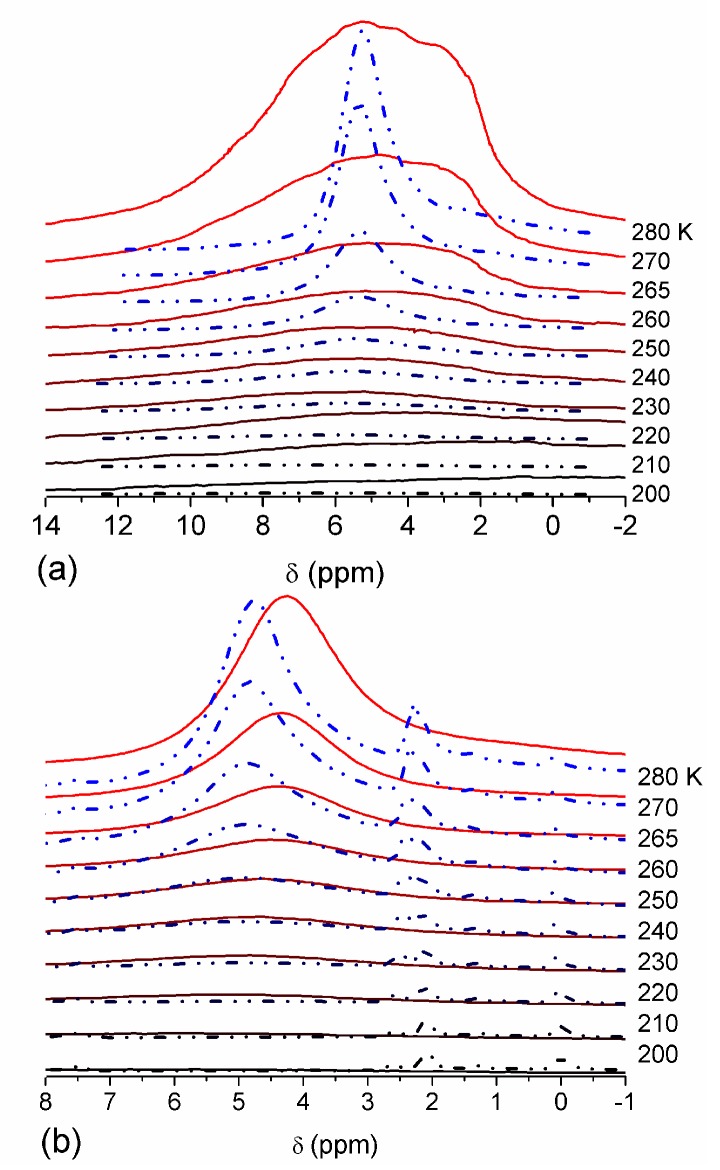
^1^H NMR spectra, recorded at different temperatures, of water bound in gelatin gel at hydration *h* = 1 g per gram of dried gelatin in different media: (**a**) air (solid lines) and C_6_D_6_:CD_3_CN = 6:1 (dashed-dotted lines), (**b**) C_6_D_6_ (solid lines) and CDCl_3_:CD_3_CN = 3:1 (dashed-dotted lines) (reproduced from Ref. [47] with permission from The Royal Society of Chemistry).

The effect of hydration on pore size distribution (PSD) of G gel was studied using low-temperature ^1^H NMR spectroscopy of freeze-dried and then re-hydrated gelatin samples. As expected, the freeze-dried G gel containing a small amount of water produced a low-intensity ^1^H NMR signal (Figure 4a). Strongly (SAW) and weakly (WAW) associated waters gave rise to weak signals at δ_H_ = 4.8 and 1.3 ppm [13]. The signal of SAW water was not detected at *T* < 250 K because its major fraction is weakly bound water, WBW, which is frozen at such low temperatures. The WAW signal intensity decreased with lowering temperature, but was still observed even at 210 K. This observation indicates that a substantial fraction of WAW was strongly bound water, SBW [13]. Adding more polar d-acetonitrile to d-chloroform (Figure 4) led to the appearance of the signal of methyl groups of CH_3_CN present as an impurity in CD_3_CN, at δ_H_ = 2 ppm. This signal can overlap with a signal of water associated with polar organic solvents, ASW. Most of ASW was bound with acetonitrile-d_3_ in HO–H···NCCD_3_ complexes giving δ_H_ = 2–2.5 ppm. The signal of WAW was observed on the right wing of the CH_3_ group signal of CH_3_CN. An increase in water amount to 10 wt % (Figure 4) led to the appearance of a broad signal of strongly associated water at δ_H_ = 4.8 ppm. The intensity of SAW signal sharply decreased at lowering temperature whereas the ASW signal intensity increased. Cross-linking of G gel with glutaraldehyde reduced water bonding in comparison with unmodified gelatin [13,44,48]. A broad asymmetrical signal of SAW was observed in a more strongly hydrated G gel, in which, at *h* = 1 g/g, about 10% of the total pore volume was filled (Figure 5a). The splitting of this signal into two signals was due to the presence of several forms of SAW and spatial heterogeneity of the material. In the sample placed in C_6_D_6_ only a broad signal of SAW was observed (Figure 5) reflecting a more uniform spatial distribution of bound water due to the filling of macropores by d-benzene and changes in water location inside pores of various sizes. In a mixture of solvents, such as C_6_D_6_ + CD_3_CN or CDCl_3_ + CD_3_CN, signals of WAW (δ_H_ = 1.3 ppm), ASW (δ_H_ = 2–2.5 ppm) and CH_3_ groups of CH_3_CN (δ_H_ = 2 ppm) were observed (Figure 5). These results show that water structure and location inside pores are affected by the presence of co-adsorbates (polar or nonpolar organic solvents), their characteristics (polarity, hydrophilicity, hydrophobicity) and concentration.

**Figure 6:**
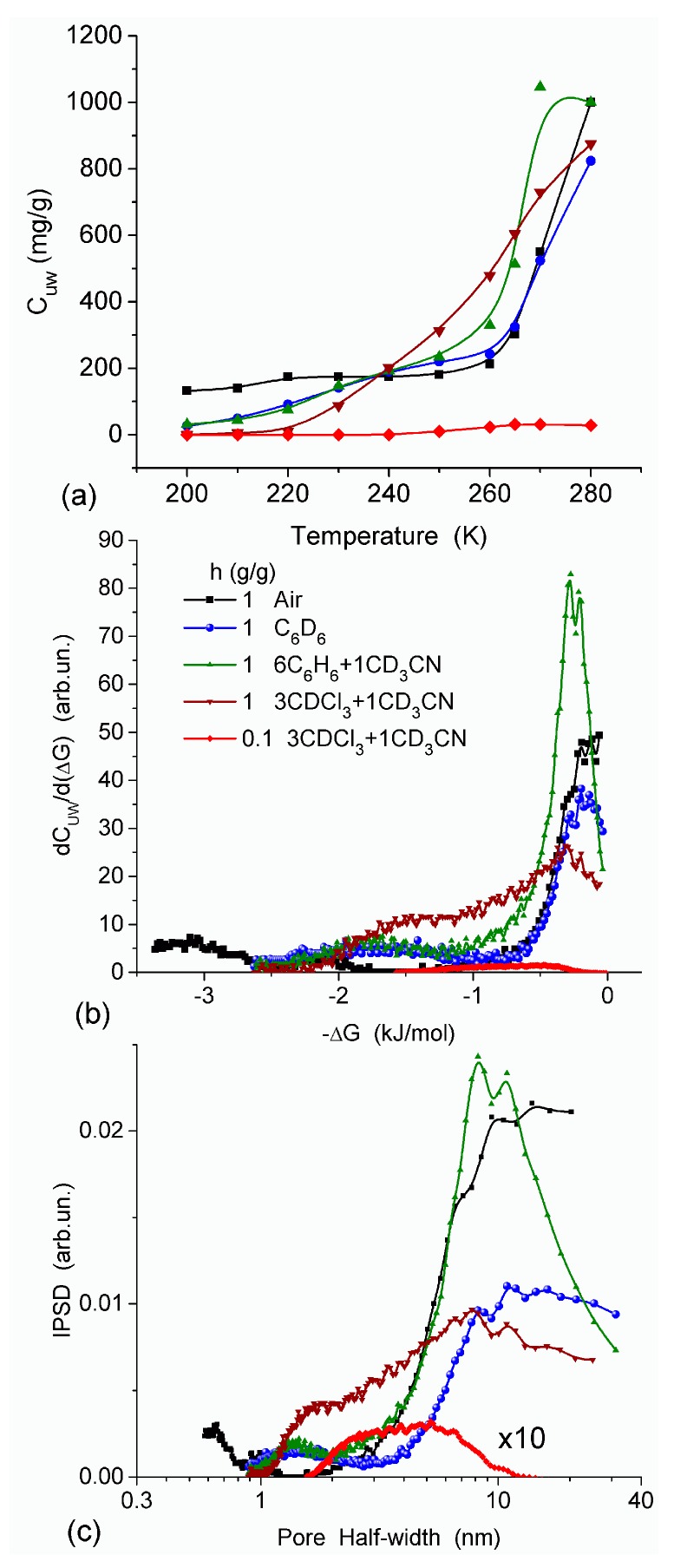
(**a**) Amount of unfrozen water (C_uw_) as a function of temperature; (**b**) derivative dC_uw_/d (ΔG), and (**c**) pore size distribution (NMR cryoporometry) for G gel in different media (reproduced from Ref. [47] with permission from The Royal Society of Chemistry).

In agreement with the DSC data, the main fraction of water bound in G gel determined by cryo-NMR, was WBW, which is due to its macroporous structure. The amount of SBW in G gel at *h* = 1 g/g corresponds to 0.2–0.3 cm^3^/g (Table 2, *V*_nn_, Figure 6). The main fraction of strongly bound water is probably located in a confined space between adjacent and cross-linked macromolecules. Organic solvents can displace this water reducing *S*_nn_ and *V*_nn_ of narrow nanopores (Table 2) and changing the size distribution of pores filled with bound water unfrozen at *T* < 273 K (Figure 6). The water located in macropores of G gel can be attributed to NBW. Therefore, the main location of water bound in G (*V*_nn_ + *V*_mn_) is likely to be in swollen walls of macropores or at their surface. The displacement of bound water from narrow nanopores by co-adsorbates (organic solvents) can be important for practical application of HG. The PSD in hydrated G gel determined by NMR cryoporometry (Figure 6c) and DSC thermoporometry is similar to that in HEMA-AGE gel (Figure 3a). This similarity of water structure in different HG is due to the presence of large amounts of bulk water masking fine effects related to bound water.

**Figure 7:**
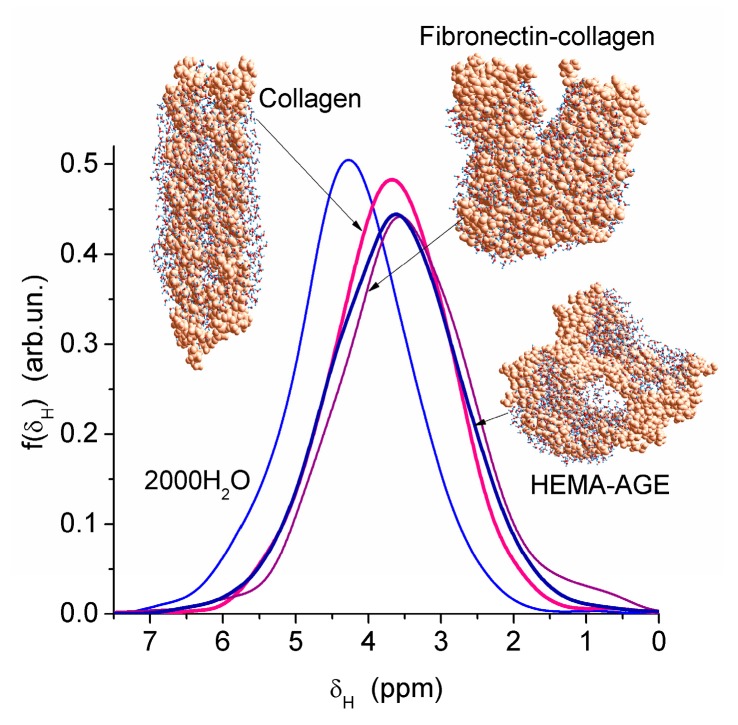
Theoretical ^1^H NMR spectra of water bound to models of partially hydrated gels with cross-linked HEMA-AGE (2373 atoms) with 1192 H_2_O, collagen (two triple coils (1639 atoms) and 1032 H_2_O) and fibronectin (8–9 Fn)—collagen (3200 atoms) with 827 H_2_O (geometry optimized with PM6 method).

Nanopores of 1–2 nm in radius were observed in areas of both low and high hydration in the HEMA-AGE model (Figure 7). The use of PM6 method for the hydrated HG fragments gave the hydration energy of macromolecules and macromolecule-macromolecule interactions. Two hydrated triple coils (i.e., six fragments) of collagen were used as a model of gelatin (Figure 7). Their hydration energy *E*_h_ was calculated to be −9.5 kJ/mol per each water molecule. This value is 3- to 4-fold smaller than the energy of a strong hydrogen bond, because a significant fraction of water molecules are located far from the protein molecules (Figure 7b). The energy of protein-protein interaction in the triple coils, *E*_pp_, is relatively high, being −19 kJ/mol per each amino acid residue. Taking into account the solvation effect it becomes much higher, −106.7 kJ/mol per each amino acid residue, because water molecules acting as bridges between neighboring chains enhance their interactions. The hydration energy *E*_h_ = −28.6 kJ/mol of the hydrated fibronectin—collagen complex is greater than *E*_h_ of collagen alone owing to more densely packed water layers in the former (Figure 7). For HEMA-AGE gel *E*_h_ is minimal (−8.6 kJ/mol) because a significant fraction of water molecules (larger than that for collagen model) is located farther from the polymer. This result is in agreement with the DSC and NMR data showing that water is weakly bound in the HEMA-AGE and gelatin hydrogels. The Gibbs free energy of bound water, Δ*G*, estimated from the NMR study, was −3.5 kJ/mol for the first layer (Figure 6b), i.e., Δ*G* > *E*_h_ (*E*_h_ ≈ Δ*H*_f_) owing to an entropy decrease in the bound water layer. The properties of bulk and bound waters, such as the activity, mobility, diffusivity, etc., strongly differ [13,14]. Since macropores are the main contributor to the HG porosity, the relative amounts of SBW + WBW in hydrogels are typically small, <10 wt % of bulk water, because most of water is located in the macropores. It is practically non-bound and behaves as bulk water.

**Figure 8:**
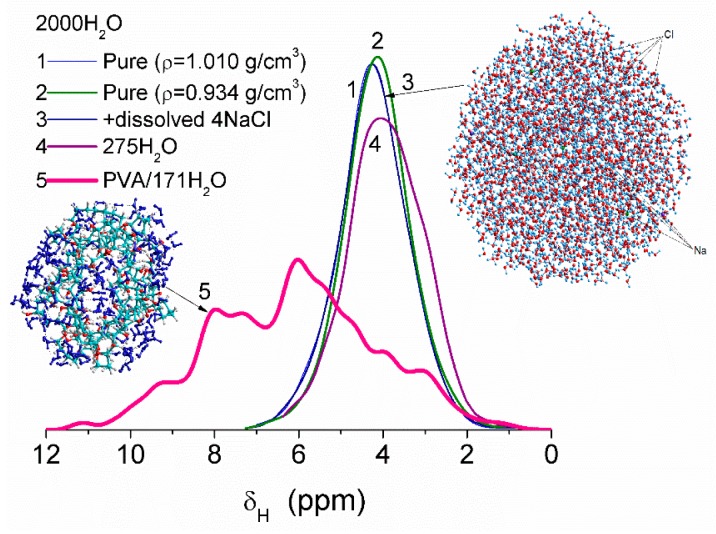
Chemical shifts of water molecules in clusters: pure (curves 1, 2, and 4), with dissolved NaCl (curve 3) and bound to PVA fragments cross-linked by glutaraldehyde (curve 5). Computational models are based on calculations using PM6 and PM7 methods and correlation functions based on DFT and PM6 or PM7 calculations of the same water clusters (adapted from [13]).

Some variation in water density due to heating or dissolving NaCl causes smaller changes in the values of δ_H_ than interaction of water molecules with fragments of PVA cross-linked by glutaraldehyde (Figure 8). This difference is due to significant changes in the organization of water molecules in the clusters upon interaction with PVA. Correlation functions between the values of δ_H_ (DFT, B3LYP/6-31G(d,p) or ωB97X-D/cc-pVDZ [52]) and atomic charges q_H_ (PM6 or PM7) were determined using several water clusters with 8, 16, and 44 molecules [13]. Some calculations of clusters were performed using WinGAMESS ver. 16.1 [53] and Firefly ver. 8.20 [54] program suites. Visualization of molecular structures was carried out using GaussView [55], Chemcraft [56], Torch [57,58], and VEGA ZZ software [59].

The water structure in HG produced from Salvia nutlets was analyzed using DSC [60]. Adding 0.1 M urea or alkali-metal salts did not influence the position or intensity of sharp endothermic peaks. The distribution of freezable (i.e., NBW + WBW) and non-freezable (SBW) waters had small effect on the order-disorder portions in the polymer network. A significant amount of freezable water appeared in HG at *h* ≥ 50 wt % [60].

The swelling behavior of a superabsorbent HG based on pectin (Pec) and polyacrylic acid (PAA) was studied [61]. Acrylic acid (AA) was graft copolymerized onto pectin backbones by a free radical polymerization technique using ammonium persulfate (APS) as initiator and *N*,*N*′-methylenebis(acrylamide) (MBA) as a cross-linker. Under the optimized conditions, the maximum capacity of swelling in distilled water was found to be 348 g/g. Absorbency of the synthesized HG was also measured in aqueous solutions of NaCl and CaCl_2_. The results indicated that the swelling ratios in comparison to pure water decreased with an increase in the ionic strength of solution. In addition, swelling capacity was studied in solutions with pH ranged from 1 to 13. The H-Pec-poly(sodium acrylate) HG exhibited a pH-responsive behavior so that pulsatile swelling-deswelling was recorded at pH 3 and 9.

Polyacrylamide hydrogels containing a mineral salt as an electrolyte are highly stretchable and transparent, which makes them interesting electrode materials for flexible electronics, but they easily dry out because of diffusion and evaporation of water, which affects their peformance and requires special storage conditions [62]. To improve capacity of water retention of polyacrylamide HG, highly hydratable salts can be introduced into the HG. These hydrogels showed enhanced water retention capacity. Both the nature of the salt species and its concentration had an effect on the water retention capacity as well as the electrical and mechanical properties of polyacrylamide HG. In terms of improving water retention capacity of the hydrogel, NaCl was the least effective, while LiCl, KAc, and MgCl_2_ were effective to a different degree. The overall performance of HG containing the latter three types of salt, improved with the increase of the dissolved salt concentration. LiCl proved to be most effective for retaining water: at the initial concentration of 12 M, over 70% of the water in PAA-LiCl hydrogel was retained even in the environment with relative humidity of only 10% RH. Apart from excellent water retention capacity, this HG also showed quite good electrical properties (high conductivity of ~10 S/m, low freezing point of ~−80 °C) and good mechanical properties (low Young’s modulus of ~3 kPa, large fracture stretch of over 20). These results could be beneficial for broadening the application fields of HG.

Collagen-based materials are widely used in tissue engineering [63,64,65]. Porous collagen-glycosaminoglycan (CG) hydrogels are already used as skin substitutes to treat thermal injuries [66] or as tissue scaffolds [67]. One of the most common CG-based wound dressing consists of collagen cross-linked with a sulfated glycosaminoglycan, chondroitin-6-sulfate [68]. The texture (affected by media) of soft matters used in tissue engineering is an important characteristic affecting their performance. The HG texture strongly depends on its interaction with water, e.g., due to strong swelling [13,69]. Macropores in CG scaffolds with size ranging from 20 to 200 μm are suitable for providing support for cell accommodation and migration and functional environments promoting their growth, and in smaller CG pores located in macropore walls, various solutes can be adsorbed and utilized as nutrients by growing cells.

Investigation of water bound in hydrogels using low-temperature ^1^H NMR spectroscopy provides useful information about their texture and nanoporosity because water properties and therefore the ^1^H NMR signal depend on the local environment, interactions of structured water with macromolecules and temperature [13,69]. NMR data [65,70,71,72,73] as well as visual images of collagen hydrogel matrices and related materials, such as gelatin and polypeptide models of collagen [74,75], showed significant changes in their 3D structure upon hydration/dehydration. Collagen retained 0.12–0.47 g of water per gram and gelatin retained 0.05–0.37 g per gram of dry matrix at a relative water pressure *P*/*P*_0_ in the range from 0.25 to 0.90 [70].

Thermally stimulated depolarization current (TSDC) method [11,12,13] was used to study the temperature behavior of water bound in a variety of HG systems. TSDC can measure the dielectric properties of water that depend on relaxation of bound (dipoles of water molecules) and mobile (protons) electrical charges, as well as the dynamics of whole polymers and their fragments [11,12,13]. Both NMR and TSDC data showed that the freezing temperature of water is affected by the mobility and state of the molecules confined inside pores. Low temperature NMR and TSDC spectra provide information on the dynamics of bound water in the closest vicinity of a solid surface or biomacromolecules. The properties of water contained in the collagen-chondroitin-6-sulfate (CG) hydrogel were studied by measuring the ^1^H NMR and TSDC signal intensity of the interfacial water remaining unfrozen at temperatures below 273 K [13]. The water and protein content calculated from the dried mass of the initial HG was found to be 98.5% and 1.5% (*w*/*w*), respectively. A freeze-dried CG was re-hydrated with water from 55.0 wt % to 95.5 wt %. Since the mass ratio of chondroitin-6-sulphate to collagen was low (between ~0.074 and 0.10) the CG material could be considered as a collagen HG [11,12,13].

**Figure 9:**
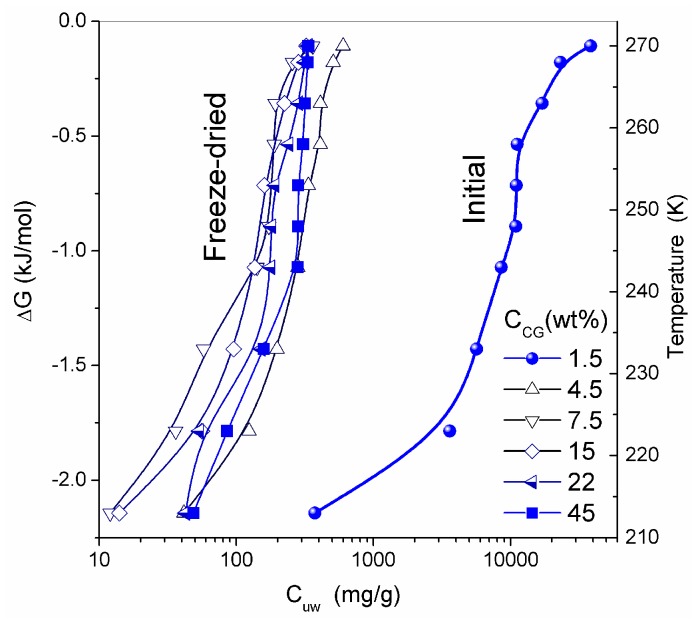
Amount of unfrozen water (*C_uw_*) as a function of temperature; and changes in the Gibbs free energy of interfacial water versus *C_uw_* at different concentrations of collagen in the hydrogel (adapted from [76] with permission, Copyright 2006, Elsevier).

SEM micrographs of dried CG and CLMS micrographs of native CG revealed the macroporous structure of the material [13]. The macropores have relatively random spherical and cylindrical-like shapes. Although the resolution of SEM and CLMS techniques is insufficient to analyze nanopores, these micrographs provide useful information on the micro- and macroporous texture in the range of pore sizes between 1 and 200 μm. At the resolution available, these pores appear to have relatively uniform PSD and thin walls [13]. Whether freeze-drying can damage the intact texture of soft hydrogels is a topic for further analysis. In general, it is considered to be a gentle procedure preserving the structural integrity of the hydrogels studied. Similar structures were observed in CSLM images for native HG. The temperature dependence of the concentration of unfrozen (*T* < 273 K) water (*C*_uw_) and the relationship between changes in the Gibbs free energy and this concentration (Δ*G* vs. *C*_uw_) are affected by interaction of water with collagen macromolecules (Figure 9). The amounts of water bound in HG (per gram of dry protein) for re-hydrated samples are significantly smaller than that in the initial CG. The lowest values of *C*_uw_^max^ and *C*_uw_^w^ were observed for HG with the highest collagen content (Table 3).

**Figure 10:**
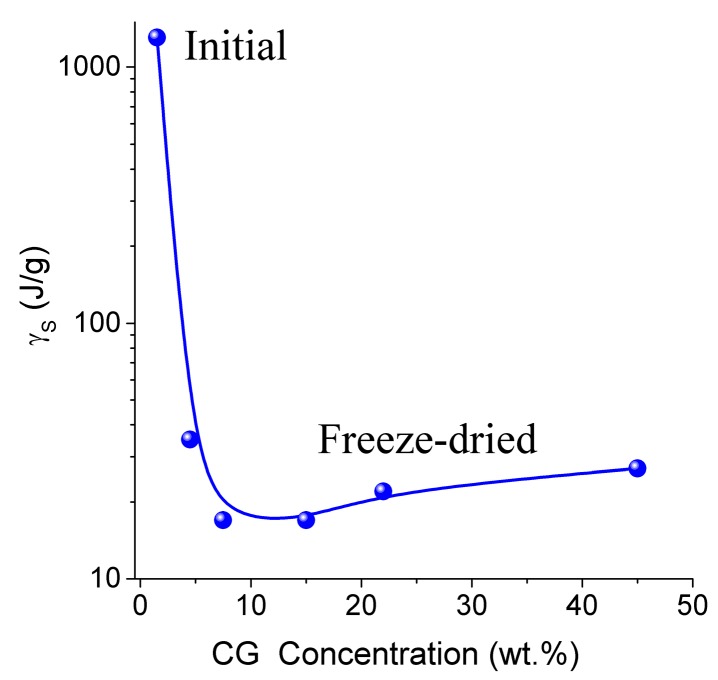
The free surface energy as a function of the collagen concentration in the CG hydrogel (adapted from [76] with permission, Copyright 2006, Elsevier).

Thermodynamic parameters of SBW (Δ*G* < −0.8 kJ/mol) and WBW (Δ*G* > −0.8 kJ/mol) were calculated from the relationship Δ*G* vs. *C*_uw_ [13,76,77]. In the NMR experiments, a fraction of SBW can remain unfrozen at *T* < 215 K. The thickness of the layers of two types of water (*C*^s^_uw_ and *C*^w^_uw_ for SBW and WBW, respectively) and the minimum of the Gibbs free energy of water caused by interactions with collagen (Δ*G*_s_ and Δ*G*_w_ for SBW and WBW, respectively) were estimated by linear extrapolation of appropriate sections of Δ*G* vs. *C*_uw_ graphs to the corresponding axis. An unusual negative correlation was found between collagen concentration (*C_CG_*) and the amount of bound water *C*^w^_uw_ (Table 3) [13]. Typically, this correlation is positive in aqueous solutions of proteins and aqueous suspensions of highly disperse oxides. This effect can be caused by a significant restructuring of the HG upon drying followed by re-hydration of the gel. The amount of WBW in CG reduced from 80 wt % to 10 wt % upon the freeze-drying and re-hydration cycle. The Gibbs free energy (γ_S_*)* dependence on collagen concentration (*C*_CG_*)* has a minimum at *C*_CG_ ≈ 10 wt % (Figure 10 and Table 3). Owing to the strongly hydrated state of the initial HG, the interfacial water layer disturbed by interactions with the collagen matrix could be as thick as ten nanometers or more. Upon removal of water during freeze-drying, distances between protein molecules shorten. This leads to ‘squeezing out’ of the bound water and decrease of γ_S_. A steep decrease in γ_S_ for re-hydrated CG is probably due to enhanced interactions between macromolecules and their aggregation at the drying step. Since these interactions are not significantly affected by re-hydration (Figure 10), this collapse of the initial hydrogel structure is irreversible due to a noticeable reduction of the contact area between water and CG. Similar behavior was shown by gelatin and albumin/nanosilica system found upon drying-re-hydration. Aggregates of 1 μm in size or larger were found in these re-hydrated materials [13].

The information obtained from TSDC measurement and the sensitivity of this method are similar to those of low-temperature ^1^H NMR spectroscopy with respect to the state of hydrogen bond network, i.e., the mobility of dipoles as bound charges or Zundel (H_5_O_2_^+^) and Eigen (H_9_O_4_^+^) cations, the spatial confinement of interfacial water domains or clusters, and the average number of hydrogen bonds per water molecule. However, the temperature range used in the TSDC measurements is broader (typically, 90–270 K) than in ^1^H NMR spectroscopy (200–280 K). Therefore combining the data obtained by two methods can provide a deeper insight into such complex systems as native, dried and re-hydrated CG [13,76].

**Figure 11:**
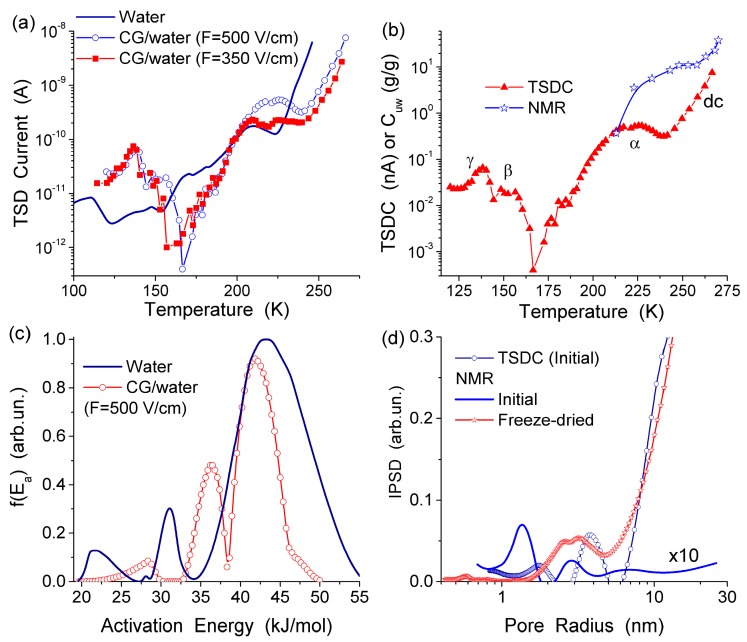
(**a**) Temperature dependence of the TSD current for the initial collagen hydrogel and “free” (bulk) water; (**b**) temperature dependences of the TSD current and the amounts of unfrozen water (*C*_uw_) (NMR) for initial collagen HG (98.5 wt % of water) (**c**) distribution function of the activation energy of relaxation in these systems; and (**d**) incremental pore size distributions for the initial CG HG calculated on the basis of ^1^H NMR and TSDC data (adapted from [76] with permission, Copyright 2006, Elsevier).

**Figure 12:**
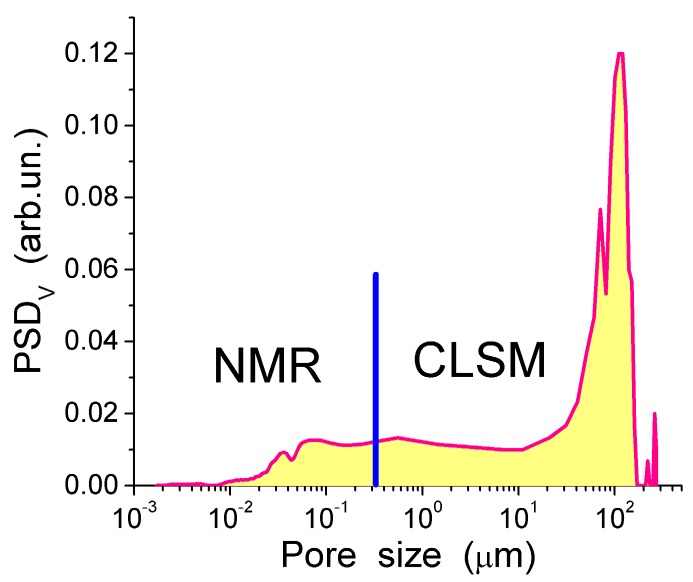
Pore size distribution calculated using ^1^H NMR cryoporometry and CLSM methods (reproduced from Ref. [44] with permission from The Royal Society of Chemistry).

The TSDC spectra (Figure 11a) and the distribution functions of the activation energy of relaxation for native CG hydrogels (Figure 11c) and pure bulk water show a significant effect that even a small amount of collagen causes on the hydrogen bond network structure of water. Variations in the polarization field (*E*_p_ = 500 and 350 V/cm) have a small effect on TSDC spectra (Figure 11a). This result confirms that the surroundings have stronger influence than *E*_p_ on the dipolar and dc relaxations of water molecules and ions in the CG HG. From the distribution of *f*(*E*) it can be concluded that most of water in this hydrogel is strongly associated. The first peak of *f*(*E*) (Figure 11c) located at low energy corresponds to molecules with the lowest number of the hydrogen bonds per water molecule. Both the first and the second peak are shifted toward higher energies in comparison with free water due to the formation of strong hydrogen bonds in the HG. This observation is in agreement with the ^1^H NMR data showing larger δ_H_ values for protons participating in the hydrogen bonds [13]. The high-energy *f*(*E*) peak corresponding to water molecules having approximately four hydrogen bonds per molecule is shifted slightly toward lower energies in comparison with free water (Figure 11c) [13,44]. This shift may be due to a certain disordering action of CG on water in the HG. According to the NMR data, about 90% of water in the hydrogel has perturbed structure. This water demonstrates enhanced, mainly rotational (since translational mobility decreases) mobility of the molecules interacting with CG which results in lowering of the freezing temperature. The results of the NMR and TSDC studies are in agreement showing a very large amount of water perturbed in the initial CG hydrogel. The results of ^1^H NMR cryoporometry and CLSM studies give the PSD in the CG hydrogel over a broad range of pore sizes (Figure 12). The amount of weakly bound water located in mesopores and freezing at temperature close to 273 K, is more than an order of magnitude larger than the amount of strongly bound water located in nanopores and freezing at *T* < 260 K. Macropores (*d* > 100 μm) and, to a lesser extent, micropores (0.1 μm < *d* < 100 μm) (non-IUPAC classification of pores) make the main contribution to the pore volume of the CG HG (Figure 12) [13,44].

Estimation of the mesh size (§) [13,44], assuming uniform CG HG structure at the protein volume fraction *v*_2,s_ ≈ 0.05, gives § ≈ 20–25 nm. This § value is in the nanopore range and much smaller than the average pore diameter *d*. The difference between the *d* and § values is due to structural nonuniformity of the CG hydrogels composed of thin pore walls (*v*_2,s_ ≈ 0.6–0.8 and § < 2–3 nm) and broad micro/macropores filled by water (*v*_2,s_ ≈ 0). Consequently, the pore walls can be impermeable for proteins and other macromolecules.

**Figure 13:**
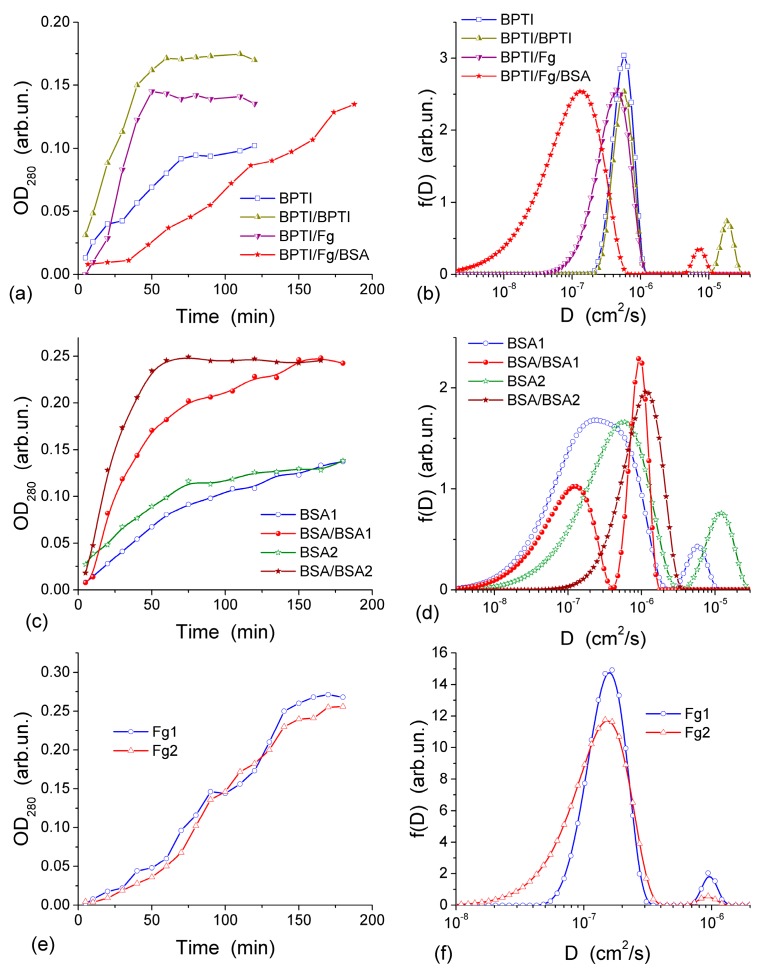
Diffusion kinetics through a collagen HG membrane (~1 mm in thickness) for (**a**) BPTI. Curve 1 is for an initial concentration of 1.23 mg/mL in the feeder cell (OD_280_ = 0.09), curves 2–4 are for an initial concentration of 2.46 mg/mL (OD_280_ = 0.18); curve 2—BPTI run after the first BPTI run; curve 3—BPTI run after Fg; curve 4—BPTI run after Fg and BSA; (**c**) BSA and BSA (with twice concentration) after the first BSA run, (**e**) Fg (initial concentration 1.7 mg/mL); curves (**b**,**d**,**f**) show the corresponding distribution functions of the diffusion coefficient *f*(*D*) for (**b**) BPTI, (**d**) BSA, and (**f**) Fg (reproduced from Ref. [44] with permission from The Royal Society of Chemistry).

The diffusion of three proteins different in size and shape, bovine serum albumin, BSA (molar mass, *m*, 67 kDa), human fibrinogen, Fg (340 kDa), and aprotinin (basic bovine pancreatic tripsin inhibitor, BPTI, 6.7 kDa) was studied in the experiments with a CG HG membrane dividing the twin-compartment cell with feeder compartment containing phosphate buffered saline, PBS, with the initial protein concentration *c*_0_ and the cell compartment containing protein-free PBS [44]. BPTI and BSA are globular proteins of different sizes while Fg is a much larger rod-like protein. These proteins can pass through a thin CG membrane (~1 mm in thickness) because the macropores in CG are much larger than the size of the proteins. The diffusion was investigated using both fresh CG and the membrane, through which the first diffusion experiment was already run using BPTI, BSA, Fg or Fg/BSA [44]. To gain more information from the diffusion kinetics data, the distribution function of the diffusion coefficient *f*(*D*) was calculated using an integral equation

(1) $$c(x,t)=\frac{c_{0}}{2\sqrt{\pi }}\int_{D_{\min}}^{D_{\max}}\frac{1}{\sqrt{Dt}}\exp(-\frac{x^{2}}{4Dt})f(D)dD,$$

where *x* is the CG membrane thickness, *D*_min_ and *D*_max_ are the minimal and maximal diffusion coefficients, respectively, and *t* is the observation time. This equation was solved using a regularization procedure CONTIN with unfixed regularization parameter as described in [44]. The highly interconnected macroporous structure of CG (Figure 11 and Figure 12) provides appropriate conditions for protein diffusion through the CG membrane. The diffusion kinetics of BPTI, BSA and Fg are shown in Figure 13 [44]. According to the experiments, within 60–65 min from the BPTI diffusion start, The BPTI system reaches equilibrium within 60–65 min (Figure 13a). The equilibrium time is affected by BPTI concentration and diffusion pre-run of proteins (Figure 13a). BSA-containing system reaches equilibrium within 75 min (Figure 13c), and Fg-containing system equilibrates within 90 min (Figure 13e). These results are characteristic of the relationship between the values of *D* for individual molecules and their hydrodynamic radius: the larger the molecules, the slower their motion (diffusion) in the solution if protein aggregation and interaction with the pore and cell walls could be ignored. This relationship can be illustrated by the values of diffusion coefficients *D*_0_ of BPTI, BSA, and Fg molecules in the aqueous solution, which are (0.8–1.9) × 10^−6^, (0.6–1.0) × 10^−6^ and (1.9–3.1) × 10^−7^ cm^2^/s, respectively [78,79,80]. The differences in the *D*_0_ values are due to much smaller (by an order of magnitude) *m*_BPTI_ than *m*_BSA_, and the latter being five times smaller than *m*_Fg_ [44].

*D*_0_ values of proteins can depend on (i) the concentration of solutes that may cause their aggregation, (ii) pH and (iii) salinity of the solution, which may influence the shape/size of protein molecules. These effects, as well as protein interactions with CG, cause broadening of the distribution function *f*(*D*) range. This range depends on the protein types. The broadening of *f*(*D*) for unimodal distributions (Figure 13b,d,f) to the lower *D*_0_ values as well as appearance of an additional peak at lower *D*_0_ (Figure 13d), indicate that the diffusion of some of protein molecules through the CG membrane is slower than in the free aqueous solution. This is most likely due to protein interactions with the macropore walls and adsorption to the CG matrix. The adsorption of proteins on the surface of transport CG macropores reduces their diameter. This phenomenon is well known as biofouling and it slows the rates of diffusion, particularly affecting narrow pores. A pre-run of Fg, and especially a pre-run of BSA and then Fg, slowed down BPTI diffusion (Figure 13b). Narrowing of transport pores caused by the protein adsorption on their walls is an obvious cause of hindering diffusion, which can be further enhanced by the interaction between the dissolved and adsorbed protein molecules. A pre-run of smaller BPTI molecules does not demonstrate such a pronounced negative effect on the second BPTI run as evidenced by the main *f*(*D*) peak remaining in the same position. An additional small peak of *f*(*D*) appearing at *D* > 10^−5^ cm^2^/s is probably due to diffusion of the BPTI molecules remaining in the membrane after the first run. Diffusion retardation of BSA is observed after the pre-run of BSA (Figure 13d) or Fg, similar to the effects of pre-adsorbed Fg and BSA on the BPTI diffusion (Figure 13b).

The appearance of several peaks of *f*(*D*) (Figure 13) reflects realization of different mechanisms of protein diffusion, which may include protein interactions with the macropore walls and with the pre-adsorbed protein layer, protein aggregation both in the solution and at the macropore walls. However, the main *f*(*D*) peaks for all samples remain close to *D*_0_ values. It means that the protein diffusion occurs through macropores (characterized by *D*_por_) of the CG membrane at the rate similar to the diffusion of individual molecules in the aqueous solution and *D*_por_ ≈ *D*_0_. Consequently, NBW in macropores of CG HG has the properties similar to that of free bulk water.

**Figure 14:**
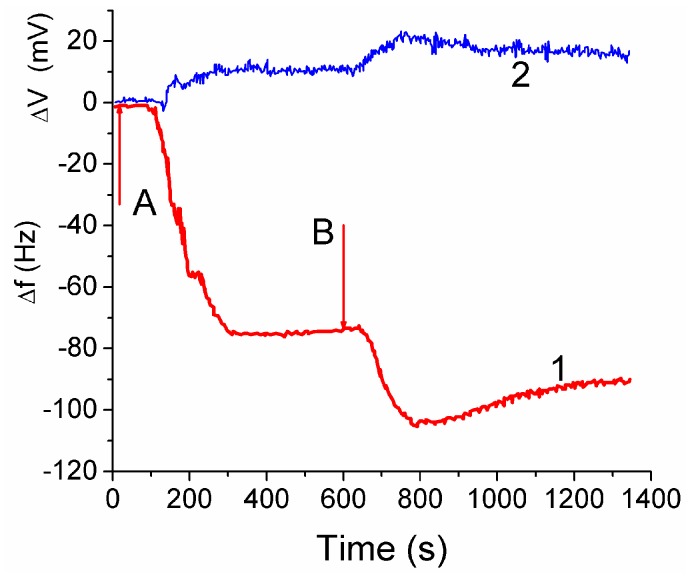
Changes in frequency (1) and auto-gain controller voltage (2) on two injections (A and B) of 0.1 mL aliquot of 3T3 fibroblast suspension (2.0 × 10^6^ cell mL^−1^) upon an unsupported section of CG HG laid the surface of a 10 MHz gold coated crystal. Flow injection rate 0.01 mL min^−1^, 37 ± 0.1 °C, pH 7.2, PBS (reproduced from Ref. [44] with permission from The Royal Society of Chemistry).

The attachment of fibroblast cells to CG HG was monitored by grafting a layer of the hydrogel onto a surface of a quartz crystal microbalance (QCM) sensor. It led to reduction of the resonant oscillation frequency by Δ*f*_1_ = −75 Hz after the first injection of cell suspension (Figure 14, curve 1 at *t* < 600 s). After the second injection the total Δ*f*_12_ ≈ −105 Hz, i.e., |Δ*f*_2_| < |Δ*f*_1_|. The enhancement of the auto-gain controller voltage (Figure 14, curve 2) indicated cell interaction with the hydrogel. The frequency shifts Δ*f* are caused by mass addition to the sensor surface [13,44]

(2) $$\Delta f=-f_{0}^{1.5}{\left(\frac{\eta_{l}\rho_{l}}{\pi \mu_{q}\rho_{q}}\right)}^{0.5}-\frac{2f_{0}^{2}\rho_{f}h_{f}}{{\left(\mu_{q}\rho_{q}\right)}^{0.5}},$$

where Δ*f* is the measured frequency shift, *f*_0_ is the resonant frequency of the unloaded crystal, *ρ_l_* and *η_l_* are the density and viscosity respectively of the liquid in contact with the coated crystal surface, *ρ_q_* and *μ_q_* refer to the specific density and the shear modulus of quartz.

Treatment of the data shown in Figure 14 with Equation (2) gives *h*_f_ ≈ 5 nm. Assuming the size of cells to be ~10 μm, the surface coverage of CG with fibroblast cells can be calculated. This area corresponded approximately to 0.05% of the total area estimated from microscopic and NMR studies. The low coverage is due to a short time (~20 min) of the contact between the cell suspension and CG in the QCM experiment, which is insufficient for cell migration into the CG matrix [44].

The QCM response depends also on: (i) the dissipation of energy within the system, and (ii) changes of the viscoelastic properties of the collagen matrix and cellular layers upon changes in the surroundings. The QCM measurements showed that fibroblast cells interaction with the CG HG occurred relatively quickly because the QC frequency minima were observed within first 3–5 min [44]. Subsequent decrease in the |Δ*f|* value by 13% after the second minimum is probably due to partial detachment of the cells by the liquid flow. The QSM response is affected by the water contained in the hydrogel because the increased amount of bound water results in increasing value of Δ*f*. The amount of water bound to the matrix should be accounted for in Equation (2).

## 3. Water Bound in Polymer-Nanofiller Systems

Nanocomposite hydrogels retain 3D polymeric networks in the presence of nanoparticles or nanostructures [13]. Nanoparticles of a variety of materials (polymeric, carbon-based, metallic, ceramic, clays, fumed oxides) can be incorporated within the HG network to obtain reinforced nanocomposites. Nanocomposites represent a new class of materials with properties typically non-characteristic for the individual components [81].

Competitive interactions of macromolecules with a particle surface, water as a solvent and neighboring macromolecules were studied [13]. These interactions do not cause very large changes in the Gibbs free energy (Δ*G*) of composites. However, these changes depend on features of interactions of charged, polar, weakly polar and non-polar functionalities with solvent or solute molecules/ions, as well as changes in the organization of solvent and adsorbate molecules in the adsorption layer. For instance, −Δ*G* for macromolecules is in the range of 5–16 kJ/mol (strong PVA-PVA interactions due to O–H⋅⋅⋅O hydrogen bonds), 1–5 kJ/mol for fibrinogen (great desolvation energy as a destabilizing factor vs. strong hydrogen bonds as a stabilizing factor), 5–25 kJ/mol for PVP (weak PVP–PVP interactions due to the absence of proton-donor functionalities in PVP) and 5–35 kJ/mol for polyoxyethylene, POE (very weak POE–POE interactions due to absence of the proton-donor groups) [13].

**Figure 15:**
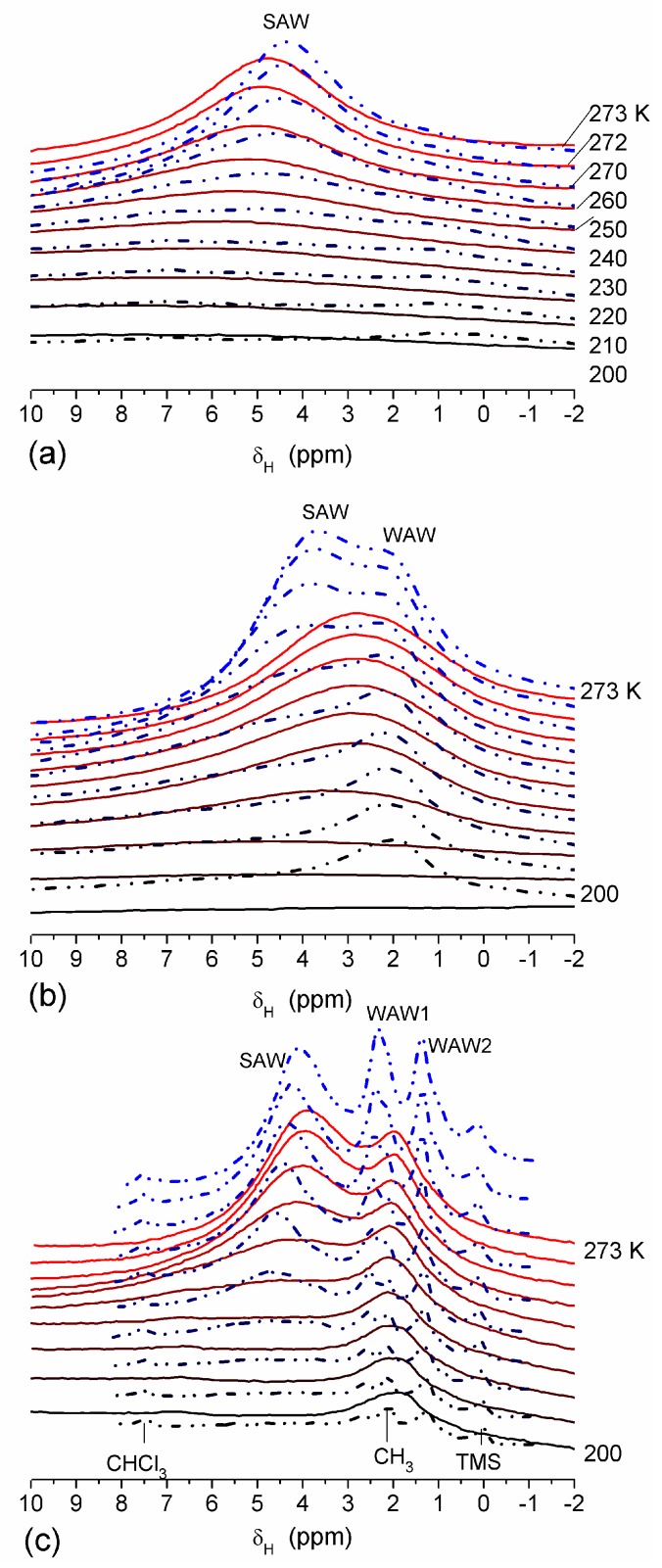
^1^H NMR spectra of water bound to (**a**–**c**) CM1 and (**c**) CM2 (dot-dashed lines) at hydration *h* = 2.3 wt % in different media: (**a**) air (solid lines) CDCl_3_ (dot-dashed lines), (**b**) CD_3_CN (solid lines) CD_3_CN:CDCl_3_ = 1:2.6 (dot-dashed lines), (**c**) CD_3_CN:CDCl_3_ = 1:5.

The features of SBW and WBW were studied in composites containing low amounts of water [13]. The interfacial behavior of water was analyzed in composites of hyaluronic acid (HA) with silica A-300 (fumed silica at *S*_BET_ ≈ 280 m^2^/g) [13]. Two HA/A-300 composite materials (CM) were prepared using low (CM1) and large (CM2) amounts of water and then dried. The ^1^H NMR spectra of water at low load (2.3 wt %) bound to CM1 and CM2 in different media (Figure 15) confirmed that HA was much more uniformly distributed in CM2 than CM1 because the spectra in the former composite had a fine structure. WAW was observed in composites immersed in weakly polar CDCl_3_ or a mixture of CDCl_3_ and CD_3_CN (Figure 15, Table 3). However, in CM1 exposed to air only SAW was observed (Figure 15a) which comprised both SBW and WBW (Table 3, *C*_uw_^s^ and *C*_uw_^w^, respectively). These types of water were observed in all samples. Only in CM1 immersed in CDCl_3_/CD_3_CN (5:1) mixture, WAW/SBW was absent (Table 3). In a larger number of samples, the SBW content is greater than WBW content that can be caused by the hydrophilic properties of both A-300 and HA and low water content. In CDCl_3_ (Figure 15a) or CD_3_CN (Figure 15b) only a single signal was observed and WAW appeared as a shoulder (Figure 15a) or the spectra had up-field shift (Figure 15b). In the CDCl_3_ and CD_3_CN mixture, the signals of WAW and SAW were split (Figure 15) [13].

**Figure 16:**
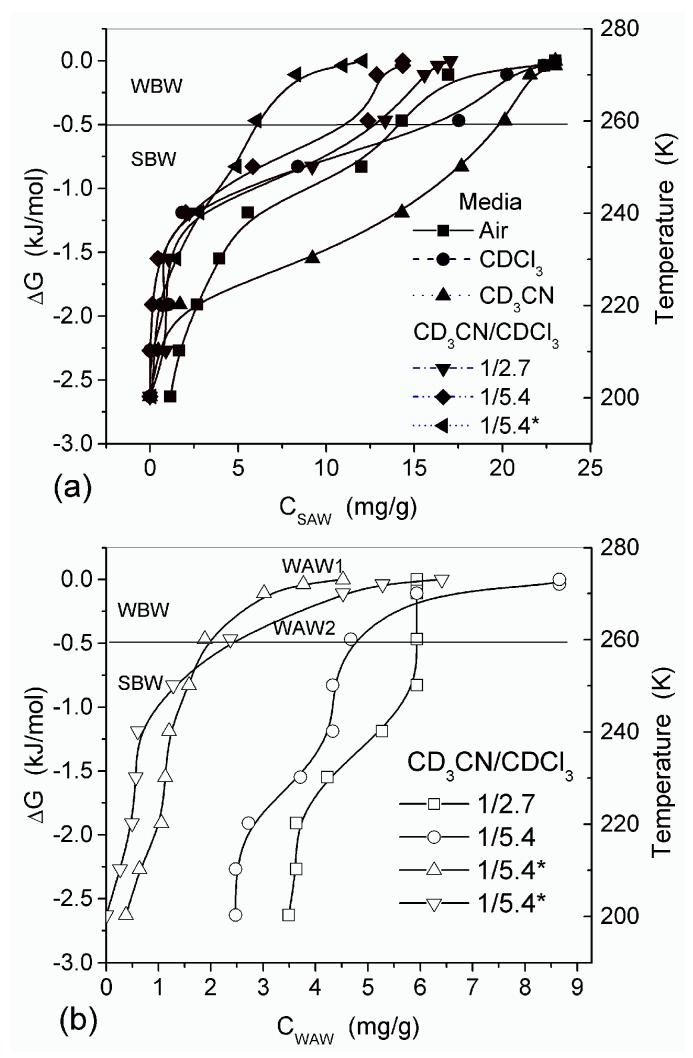
Temperature dependence of the amount of (**a**) SAW and (**b**) WAW and the relationships between changes in the Gibbs free energy and the amounts of SAW and WAW in HA/A-300 composites CM1 and CM2 (*).

**Figure 17:**
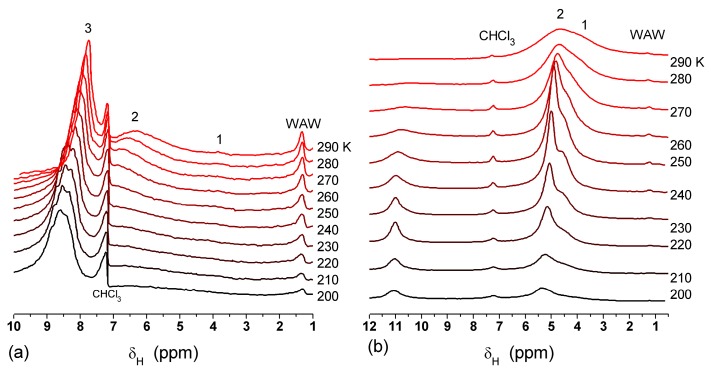
^1^H NMR spectra of CM1 with adsorbed aqueous solutions (150 mg/g) of (**a**) 18% HCl and (**b**) 16% H_2_O_2_ in CDCl_3_ medium.

The behavior of water bound in HA/nanosilica is strongly affected by the dispersion medium (Figure 16) [13]. The amount of SAW in CM2 was maximal in CD_3_CN and minimal in CD_3_CN/CDCl_3_ mixture. Fast proton exchange between water and dissolved HCl gave rise to large δ_H_ values (9–11 ppm) dependent on the acid concentration. The ^1^H NMR spectrum of HCl adsorbed on CM1 from 18% aqueous solution comprises four signals at: (i) 1.3 ppm corresponding to WAW, and (ii) three signals (nn. 1, 2 and 3) of SAW at ~4, 6–7 and 8–9 ppm, respectively (Figure 17a). The content of HCl in WAW and SAW structures differs: no HCl in WAW as indicated by the absence of signal at 1.3 ppm, no HCl in SAW which gave signal at ~4 ppm, a small amount of HCl dissolved in SAW with signal at 6–7 ppm and a more concentrated HCl solution in SAW with signal at 8–9 ppm. The intensity of signals 1 and 2 in SAW decreased with decreasing temperature, but signal 3 did not change. A fine structure of signal 3 was observed at low temperatures caused by certain nonuniformity of the composite perturbing the structure of bound SAW.

In the case of adsorption of the aqueous solution of H_2_O_2_, the ^1^H NMR spectra (Figure 17b) had certain features different from those observed for the adsorbed HCl (Figure 17a). The signal of concentrated H_2_O_2_ observed at ~11 ppm was weak, but the signal of SAW at 4–6 ppm, with small content of H_2_O_2_, had high signal intensity. WAW signal in the system with H_2_O_2_ was much weaker than WAW signal with HCl. Thus, the HCl solution in SAW tends to be more concentrated state than H_2_O_2_ at the interfaces of HA-silica composite [13].

**Figure 18:**
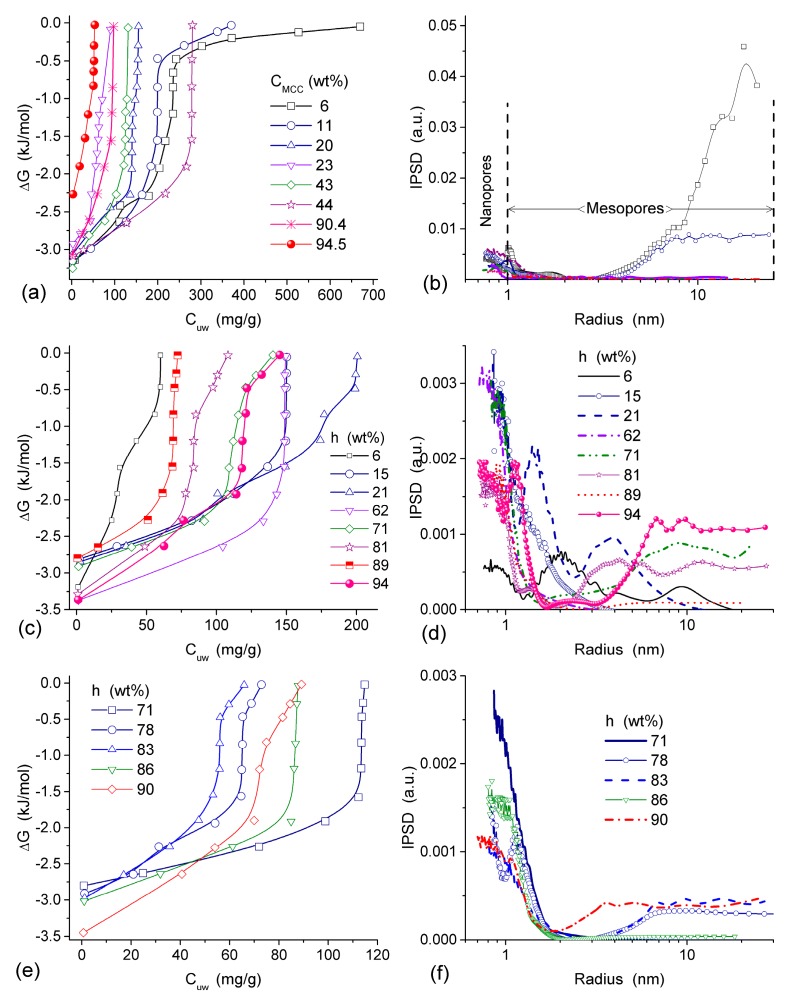
(**a**,**c**,**e**) Relationships between the amounts of unfrozen water and changes in the Gibbs free energy and (**b**,**d**,**f**) the corresponding PSD (NMR cryoporometry with GT equation at *k*_GT_ = 67 K nm) for (**a**,**b**) MCC, (**c**,**d**) MCC/A-300 (5.6:1), and MCC/TiO_2_ (3:1) (adapted from [13]).

Interaction of microcrystalline cellulose (MCC) with nanosilica A-300 (with particles of average size ~9.5 nm) and titania (with particles of average size ~100 nm) at the mass ratio 5.6:1 and 3:1, respectively, was studied using low-temperature ^1^H NMR spectroscopy in aqueous suspensions and wetted powders (Table 4 and Table 5, Figure 18) [13,82]. The vertical fragments of Δ*G*(*C*_uw_) curves in Figure 18 correspond to approximately constant concentration of unfrozen water in a wide range of Δ*G* changes meaning that adsorbed water remains unfrozen in a wide temperature range. This feature is typical of water adsorbed in nano- and/or narrow mesopores [13,83]. The presence of narrow pores filled by unfrozen water was observed in all samples studied (Figure 18b,d,f).

In MCC/oxide mixtures strong interactions of cellulose with the silica or titania surfaces result in decrease in the amounts of bound water because voids between particles, which can be filled by water, decrease in size [13,82]. Relative contributions of mesopores to the void volume decrease (Table 4 and Table 5, Figure 18). This is an expected result for soft/solid systems because of the formation of core (solid)—shell (soft, non-rigid) structures with the displacement of a significant amount of water bound to both soft and solid surfaces alone. This effect is much smaller for interactions of solid-solid particles because of the rigid particle structure of solids that leads to a small area of contacts between adjacent particles [13].

Two specific regions in the graphs of Δ*G* vs. *C*_uw_, similar to those discussed above, were found for nanosilica, PVP/silica, and ball-milled PVP/silica suspensions [13]. The first Δ*G*(*C*_uw_) region reflects a significant decline in the unfrozen water amount at temperatures near 273 K. In the other region, a slight decrease in the amount of unfrozen water causes more significant Δ*G* changes. Water in the first region is a thick layer of WBW, which is weakly perturbed by long-range intermolecular forces [13]. Water in the second region of the graph is a thin layer SBW fraction of bound water adjacent to the surface of the matrix. SBW is strongly affected by short-range forces between the surface and adsorbed molecules.

**Figure 19:**
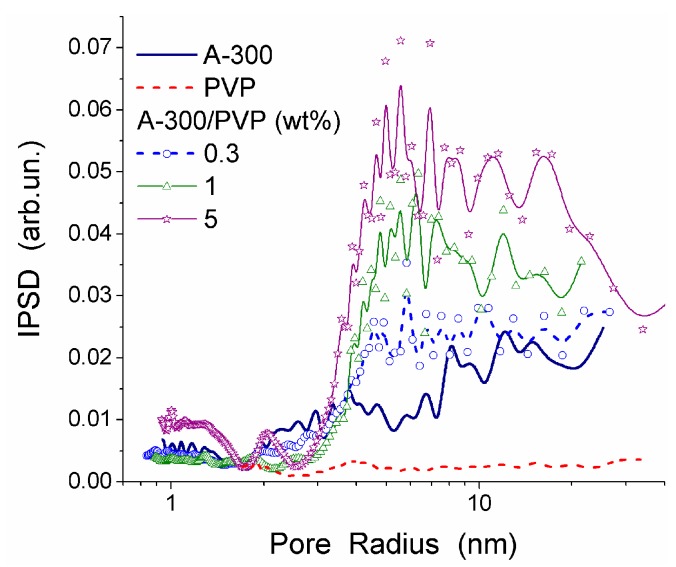
PSD (*k*_GT_ = 70 K nm) for A-300/PVP systems (adapted from [13]).

The interactions between PVP and the surface of silica nanoparticles lead to changes in the values of −Δ*G*, *C*^s^_uw_ and *C*^w^_uw_ (Table 6). An increase in *C*^w^_uw_ may be due to nonuniform electrostatic field in the interfacial layer containing tails and segments of adsorbed PVP molecules. The WBW amount in this layer is larger than in the layer surrounding polymer-free silica particles because the former is thicker than the latter (Table 6). All PVP molecules are bound to the silica surface. As a consequence, it leads to an increase in the surface area in contact with unfrozen water (Table 6, *S*_uw_). Although WBW perturbance by 0.3 wt % PVP in PBS is weaker than perturbance in the presence of 1 wt % aqueous PVP, changes in Δ*G*_w_ of this layer are similar. An increase of PVP concentration to 5 wt % causes an increase in the thickness of the unfrozen water layer and other parameters (Figure 19, and *S*_uw_, *S*_nano_, *C*^s^_uw_ *+ C*^w^_uw_ and γ_S_ in Table 6). The PSDs in Figure 19 reveal voids formed by silica nanoparticles and PVP. They are filled by unfrozen water and correspond to the mesoporous range. This is typical for the aqueous suspensions of nanosilica and other nanooxides [13].

Chitin macromolecules have been shown to stabilize graphite-hydrogel composite due to their location between the graphite particles [84]. There is a problem related to the accessibility of pores of filler particles, which are embedded into the cross-linked polymer network of hydrogels or cryogels, for target solutes [85,86,87]. This is due to, at least, two effects. First, upon cross-linking of macromolecules a part of the corresponding reagent (e.g., glutaraldehyde) can fill pores and form oligomers there, which then prevent adsorption of solutes. Second, polymers, both non-cross-linked and cross-linked can penetrate into pores or block the entrances into pores that especially occurs if the amount of porous particles is much smaller than that of the polymer. Clearly, both effects lead to reduction of the pore volume, specific surface area and amounts of SBW and WBW, but the amount of free water in the hydrogel increases [85,86,87].

## 4. Conclusions

Analysis of the state and amounts of strongly and weekly bound and non-bound waters provides valuable information for assessing various parameters of hydrogels, because water in different states are located in different pores, voids, channels of macroporous HG with porous walls (having nanopores and micropores) of marcopores. Therefore, investigations of the states of water bound in HG give additional information on changes in the structure of HG upon changes in the degree of hydration and swelling. ^1^H NMR spectroscopy, TSDC, XRD, DSC, diffusion kinetics, QCM, microscopic and other physical and physicochemical methods showed that hydrogels contain a large amount of water existing in different states. Re-hydration of the dried gels could not restore the amount of strongly bound and weakly bound waters. This effect could be explained by structural changes in the 3D network of dried HG, which is rather negative one. In the case of collagen HG, freeze-drying of the initial hydrogel causes the formation of additional collagen-collagen bonds, which are not destroyed in the re-hydration step, and this leads to the reduction of the amount of bound water and a decrease in the absolute value of free surface energy measured by ^1^H NMR. However, in the case of strongly cross-linked HG, freeze-drying weakly affects the gel structure and its properties, such as swelling degree. Thus, structural features of HG depend not only the type of the polymeric matrix but also on the cross-linking degree and porosity of the material including nano- and micropores (according to Life Science classification of pores) in the macropore walls.

Analysis of the state and amounts of unfrozen water as functions of temperature below the normal freezing point of water allows studying the nanoporous structure of walls of macropores in hydrogels. Estimation of the temperature dependence of the size and volume of nanopores filled with unfrozen water (both SBW and WBW) shows that short-range order in freeze-dried gels changes insignificantly in comparison with long-distance order. The approaches based on ^1^H NMR, DSC and TSDC methods combined with freezing-out of bulk water and comparing the structural features of the unfrozen water with its thermodynamic properties and structural characteristics provide important information on the whole porous structure of polymeric or composite HG whose properties can change upon hydration/dehydration and heating.

Studies of the diffusion of various solutes of low- and high-molecular weight in macropores of HG show that the state of water in macropores is similar to that of free water. However, interactions of solutes with the macropore walls leading to adsorption or even their penetration into the pores result in changes not only diffusion of solutes but also the behavior of water, since contributions of NBW, WBW, and SBW change and these effects depend strongly on temperature.

Thus, the organization of water bound in hydrogels depends on many factors, and comprehensive analysis of the temperature and interfacial behaviors of the water provides a deeper insight into the interfacial phenomena occurring in hydrogels at different degrees of hydration, various temperatures, at the presence of different solutes.

## Figures and Tables

**Table 1:** Characteristics of the porous structure of HEMA-AGE gel samples A, B, C and D.

Sample	A *	A ^§^	B *	C *	D *
Porosity, %	91	88	91	82	68
Surface area *(S)*, μm^2^/μm^3^	0.045	0.10	0.06	0.095	0.186
Wall thickness *(t)*, μm	9.6 ± 0.8	10.9 ± 1.6	5.7 ± 0.5	8 ± 1	9.5 ± 1.3
Pore size *(d)*, μm	64 ± 2	56 ± 1.5	47 ± 2.8	41.4 ± 6	25.7 ± 1

* CLSM image analysis. **^§^** MPM image analysis.

**Table 2:** Structural characteristics of gelatin gel based on NMR cryoporometry.

*h* (g/g)	Medium	*S* (m^2^/g)	*S*_nn_ (m^2^/g)	*S*_mn_ (m^2^/g)	*S*_bn_ (m^2^/g)	*V*_nn_ (cm^3^/g)	*V*_mn_ (cm^3^/g)	*V*_bn_ (cm^3^/g)	γ_S_ (J/g)
1	Air	465	423	43	0	0.157	0.431	0	34.4
1	C_6_D_6_	133	94	38	1	0.045	0.520	0.014	28.3
1	C_6_D_6_ + CD_3_CN	176	88	87	1	0.042	0.928	0.010	37.6
1	CDCl_3_ + CD_3_CN	84	10	74	1	0.005	0.732	0.009	35.9
0.1	CDCl_3_ + CD_3_CN	8	0	8	0	0	0.031	0	1.3

γ_S_ is the modulus of total interfacial Gibbs free energy, *S* the surface area, *V* the pore volume, h the degree of hydration.

**Table 3:** Characteristics of water bound to CM1 (samples 1–5) or CM2 (6) at fixed hydration (*h* = 23 mg/g) in different media.

Medium	Water Type	Δ*G*_s_ (kJ/mol)	*C*_uw_^s^ (mg/g)	*C*_uw_^w^ (mg/g)	γ_S_ (J/g)
Air	SAW	−3.5	15	8	1.1
CDCl_3_	SAW	−2.8	17	6	0.94
CD_3_CN	SAW + WAW	−2.7	20	3	1.61
CD_3_CN/CDCl_3_ 1:2.6	SAW	−2.2	14	0.75	0.62
	WAW	−10.0	4.5	5	1.1
CD_3_CN/CDCl_3_ 1/5	SAW	−2.7	12	11	0.81
	WAW	−7.0	0	11	1.1
CD_3_CN/CDCl_3_ 1/5	SAW	−2.6	6	6	0.45
	WAW1/WAW2	−3.0/−2.4	2/2	4.7/4.7	0.17/0.19

**Table 4:** Characteristics of water bound to MCC in aqueous suspensions and hydrated powders [13].

*C*_MCC_ (wt %)	−Δ*G*_s_ (kJ/mol)	*C*_uw_^s^ (mg/g)	*S*_uw_ (m^2^/g)	*S*_nano_ (m^2^/g)	*S*_meso_ (m^2^/g)	*V*_uw_ (cm^3^/g)	*V*_nano_ (cm^3^/g)	γ_S_ (J/g)	γ_S_ (mJ/m^2^)
6	3.08	230	338	288	50	0.567	0.126	37.5	111
20	3.01	160	235	218	17	0.155	0.098	21.1	132
43	3.17	125	230	210	19	0.131	0.092	18.6	81
94.5	2.37	52	9	0	9	0.054	0.0	4.9	

**Table 5:** Characteristics of water bound to MCC/A-300 (1–8 samples) or MCC/TiO_2_ (9–13 samples) at different water contents (*h*) [13].

*h* (wt %)	−Δ*G*_s_ (kJ/mol)	*S*_uw_ (m^2^/g)	*S*_nano_ (m^2^/g)	*S*_meso_ (m^2^/g)	*V*_uw_ (cm^3^/g)	*V*_nano_ (cm^3^/g)	γ_S_ (J/g)	γ_S_ (mJ/m^2^)
6	3.14	55	48	7	0.060	0.021	6.3	115
71	2.88	162	153	9	0.137	0.070	16.2	100
94	3.25	176	168	8	0.143	0.071	17.6	100
71	2.78	138	111	27	0.115	0.052	14.8	107
83	2.94	66	62	4	0.065	0.028	7.5	113
90	3.31	120	115	5	0.088	0.049	11.2	93

**Table 6:** Parameters of interfacial water for pure silica and silica/PVP suspensions (*C*_SiO2_ = 6 wt %).

System	−Δ*G_s_* (kJ/mol)	−Δ*G*_w_ (kJ/mol)	*C*^s^_uw_ mg/g	*C*^w^_uw_ mg/g	γ_S_ mJ/m^2^	*S*_uw_ (m^2^/g)	*S*_nano_ (m^2^/g)
SiO_2_	2.76	1.3	700	700	257	297	171
^a^ SiO_2_ + 0.3 wt % PVP	2.99	1.0	500	900	184	389	264
1 wt % PVP	1.64		300			30	0
SiO_2_ + 1 wt % PVP	2.89	0.9	520	1600	195	347	158
SiO_2_ + 5 wt % PVP	2.75	1.0	1100	1400	270	465	256

^a^ In PBS.
